# Real‐Time and High‐Resolution NIR‐II‐L Imaging of Netrin‐1‐Mediated Neurovascular Coupling Driven by Dose‐Dependent Electroacupuncture

**DOI:** 10.1002/advs.202520847

**Published:** 2026-01-27

**Authors:** Yicong Wang, Zi‐Han Chen, Jiajia Li, Wei Hu, Ying Cao, Yu Wang, Xiaoyu Tong, Wenhan Lu, Yuze Yang, Yuehao Wang, Yan Xiao, Wenhao Gao, Yuning Chen, Yuyan Hou, Fan Zhang, Yi Feng

**Affiliations:** ^1^ Department of Integrative Medicine and Neurobiology School of Basic Medical Sciences Shanghai Medical College Brain Science Collaborative Innovation Center State Key Laboratory of Medical Neurobiology Fudan University Shanghai China; ^2^ Shanghai Key Laboratory of Acupuncture Mechanism and Acupoint Function Shanghai Institute of Acupuncture and Moxibustion Shanghai China; ^3^ Fudan Zhangjiang Institute Shanghai China; ^4^ Department of Chemistry College of Smart Materials and Future Energy New Cornerstone Science Laboratory State Key Laboratory of Molecular Engineering of Polymers Shanghai Key Laboratory of Molecular Catalysis and Innovative Materials and Ichem Fudan University Shanghai China; ^5^ Suzhou TCM Hospital Affiliated to Nanjing University of Chinese Medicine Gynecology Department Suzhou City Jiangsu Province China; ^6^ Department of Neurology Affiliated Hospital of Shandong University of Traditional Chinese Medicine Jinan China; ^7^ Department of Ophthalmology & Visual Science Eye & ENT Hospital Shanghai Medical College Fudan University Shanghai China; ^8^ Department of Medical Physics Shanghai Proton and Heavy lon Center Shanghai China; ^9^ Shanghai Key Laboratory of Radiation Oncology Shanghai China; ^10^ Shanghai Engineering Research Center of Proton and Heavy Ion Radiation Therapy Shanghai China; ^11^ Reproductive Medicine Center Zhongshan Hospital Fudan University Shanghai P. R. China

**Keywords:** NIR‐II‐L imaging, optical theranostics, ovarian neurovascular coupling, real‐time imaging

## Abstract

Accumulating evidence implicates that disrupted ovarian neurovascular coupling is a vital driver for polycystic ovary syndrome. Electroacupuncture (EA), as a form of peripheral neuromodulation, offers a potential therapeutic avenue to restore this coupling and improve ovarian function. However, how neural regulation leads to vascular changes remains unclear. Conventional histological and imaging methods cannot capture either immediate or long‐term effects of EA on neurovascular dynamics, as they are limited by the lack of real‐time and continuous in vivo visualization. Here, core–shell lanthanide nanocrystals α‐NaYbF_4_:2%Er,2%Ce@NaYF_4_ showed deep‐tissue penetration in the second near‐infrared long‐wavelength region (NIR‐II‐L, 1500–1900 nm). Moreover, it could achieve real‐time and high‐resolution visualization of EA‐induced structural and functional changes in the ovarian blood vessels. Our results showed that immediate EA (iEA) at specific parameters and acupoints transiently induced ovarian vasodilation, while cumulative EA (cEA) further strengthened sympathetic‐vascular coupling, leading to sustained improvements in local perfusion and follicular development. Additionally, we validated that these effects were dependent on the neurovascular coupling mediator Netrin‐1, an axon guidance molecule increasingly recognized for its role in arterial innervation and blood flow regulation in peripheral organs. This study elucidated parameter‐specific mechanisms by which EA regulated ovarian function and proposed a visualizable strategy for in vivo analysis of neurovascular structural‐functional remodeling.

## Graphical abstract

Disrupted neurovascular coupling contributes to ovarian dysfunction in polycystic ovary syndrome. Using near‑infrared II long‑wavelength imaging, this study visualizes how electroacupuncture dynamically regulates ovarian blood vessels in vivo. Immediate and cumulative stimulation elicit distinct vascular responses via a Netrin‐1 dependent mechanism that reveals a neuromodulatory pathway linking neural input to vascular remodeling and follicular improvement.

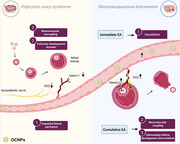

## 1 Introduction

Polycystic ovary syndrome (PCOS) is a common endocrine and metabolic disorder, affecting approximately 10–13% of women of reproductive age worldwide [1, 2, 3]. As a non‐pharmacological intervention, acupuncture has demonstrated efficacy in improving ovulatory dysfunction, insulin resistance, and weight management. Consequently, it has been incorporated into recommendations within several clinical practice guidelines [4, 5, 6]. Electroacupuncture (EA), as a form of peripheral somatic stimulation, has been shown to remotely modulate the function of specific visceral organs by activating somatovisceral reflex pathways, exhibiting site dependency and organ specificity [7, 8, 9]. For instance, studies utilizing laser Doppler flowmetry have demonstrated that EA can rapidly enhance local ovarian perfusion, indicating its potential to regulate vasodilation through neurogenic mechanisms [10]. In the central nervous system, EA has been shown to activate specific sub‐populations of somatosensory neurons, which subsequently drive cholinergic neurons in the dorsal motor nucleus of the vagus (DMV). This activation initiates vagovagal reflexes to modulate gastrointestinal and adrenal functions, revealing a functional coupling pattern characterized by ‘molecularly defined inputs and structurally specified outputs [7].

In the peripheral sympathetic nervous system, the ovary, as a deep internal reproductive organ, is primarily innervated by the superior ovarian nerve and the celiac ganglion. Anatomical studies have demonstrated that sympathetic nerve terminals closely surround the ovarian hilum, adjacent to the main vasculature and capillary network, forming highly specialized neurovascular regulatory units [10, 11, 12]. These specialized structures play a critical role in regulating follicular development, steroidogenesis, and ovulation [13, 14, 15, 16]. The EA‐induced changes in ovarian microcirculation may depend on structural coupling between specific sub‐populations of sympathetic axons and vascular endothelial cells. However, critical nodes in this regulatory axis, particularly whether functionally specific neurovascular connectivity exists, remain unknown. Additionally, current research predominantly relies on endpoint measurements or ex vivo tissue analysis, lacking in vivo, continuous, and quantifiable methods for monitoring dynamic physiological responses. Consequently, the key regulatory nodes mediating neurovascular signaling are still poorly elucidated [17, 18, 19, 20]. Notably, the therapeutic efficacy of EA heavily depends on stimulation parameters, including frequency, acupoint selection, and duration [21, 22]. This ‘mechanistic invisibility’ not only constrains the theoretical advancement of acupuncture but also impedes the optimization and clinical translation of individualized stimulation parameters.

Due to the deep intra‐abdominal location and complex vascular architecture of the ovary, in vivo imaging remains technically challenging in terms of penetration depth, spatial resolution, and dynamic acquisition. Existing imaging modalities provide important insights but are suboptimal for real‐time vascular imaging in deep visceral organs. Photoacoustic imaging improves penetration and enables hemoglobin‐based functional contrast, yet is constrained by depth‐resolution trade‐offs and system complexity for high‐resolution, organ‐scale imaging [23]. In comparison, multiphoton microscopy achieves high cellular resolution. Still, it is fundamentally limited by tissue scattering, restricted field of view, and the need for optical access, hindering continuous imaging in deep organs [24].

In this regard, the second near‐infrared (NIR‐II, 1000–2000 nm) fluorescence, especially NIR‐II long‐wavelength region (NIR‐II‐L, 1500–1900 nm), has been proven to offer high signalto‐noise ratio and deep‐tissue penetration for in vivo bioimaging. With the development of lanthanide nanocrystals, NIR‐II‐L region has recently been widely applied to in vivo cerebral vascular imaging, enabling dynamic tracking of neurovascular interactions at the capillary level [25, 26].

In this study, through NIR‐II‐L emitted α‐NaYbF_4_:2%Er,2%Ce@NaYF_4_ core–shell lanthanide nanocrystals, an integrated research framework was developed for investigating EA‐induced remodeling of the ovarian neurovascular microenvironment in PCOS models. Real‐time and high‐resolution NIR‐II‐L imaging could achieve dynamic monitoring of EA‐induced microvascular dilation in the ovary, comparing response patterns between immediate and cumulative stimulations. In addition, 3D tissue clearing and molecular interventions were employed to provide structural and mechanistic insights. The detailed architecture of the sympathetic nerve‐vascular‐follicular tripartite network was systematically mapped. These results revealed that tyrosine hydroxylase‐positive (TH^+^) sympathetic axons distribute along ovarian blood vessels and form synapse‐like contacts with endothelial cells. Based on these observations, we established a co‐culture system using the superior cervical ganglion and ovarian vascular adventitia. Moreover, through Netrin‐1 pathway blockade experiments, we validated its essential role in mediating neurovascular interactions and the vascular response to EA. These findings establish a mechanistic and visual paradigm for decoding EA‐induced neurovascular plasticity in reproductive organs, bridging traditional stimulation with modern systems‐level understanding of ovarian function.

## 2 Results

### 2.1 NIR‐II‐L Imaging Reveals iEA‐Induced Ovarian Vasodilation

**FIGURE 1:**
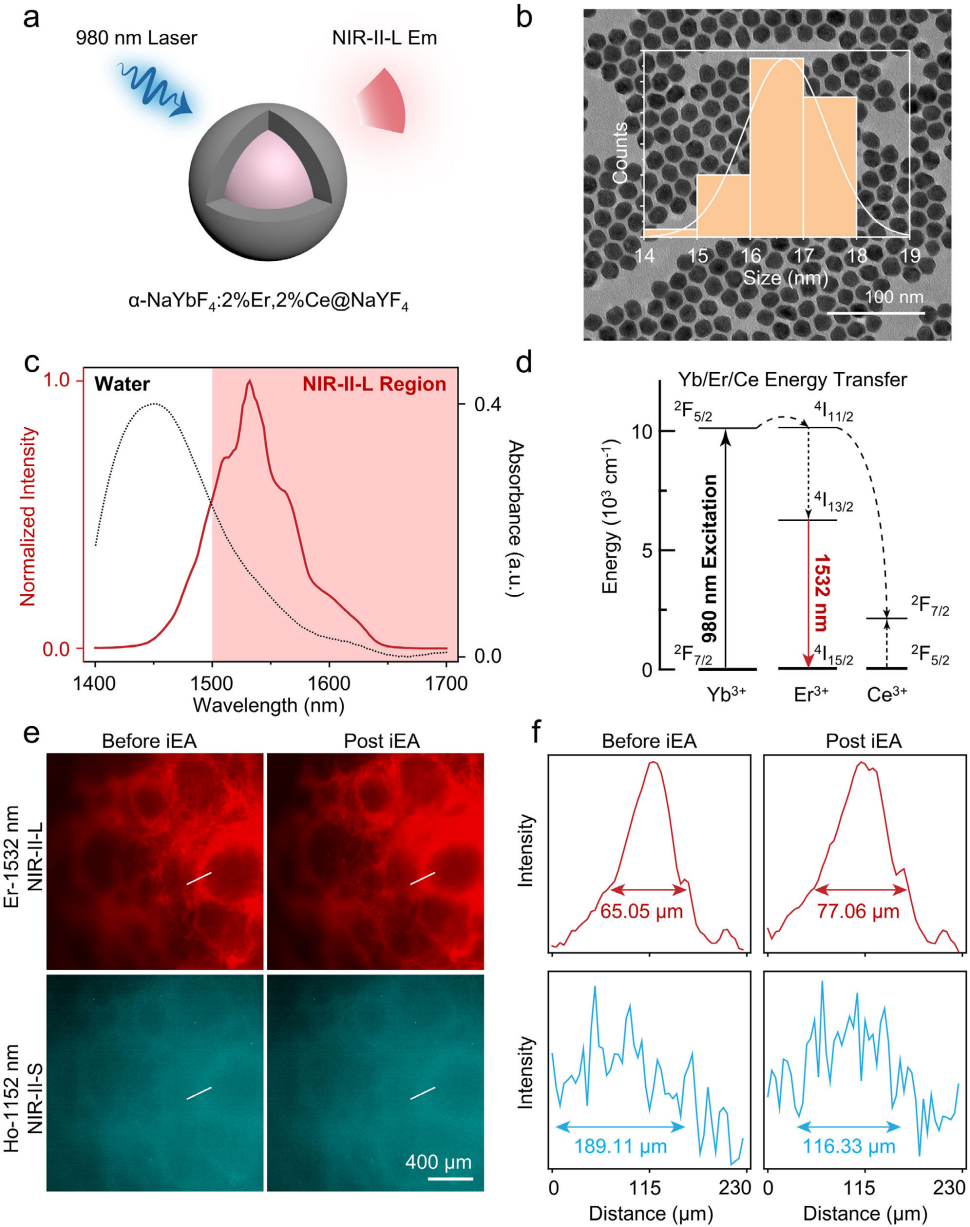
Structural and optical properties of DCNPs and NIR‐II bioimaging in the ovary. (a) Schematic illustration of α‐NaYbF_4_:2%Er,2%Ce@NaYF_4_ core–shell lanthanide nanocrystals. Such nanocrystal could emit fluorescence within NIR‐II‐L region under 980 nm excitation. (b) TEM images and relative size distribution of as‐synthesized DCNPs. It showed uniform sphere shape with average size about 16.67±0.78 nm. (c) Near‐infrared fluorescent spectrum of the as‐synthesized DCNPs under 980 nm (red line) and water absorption curve (black line) from 1400 to1700 nm. (d) The fluorescent mechanism and the energy transfer process of the as‐synthesized DCNPs. (e‐f) NIR‐II bioimaging of ovarian region by usage of two lanthanide nanocrystals with different emission wavelength, and corresponding cross‐section intensities grabbed from the white line in (e). In vivo bioimaging showed superior FWHM in NIR‐II‐L region than NIR‐II‐S region.

**FIGURE 2:**
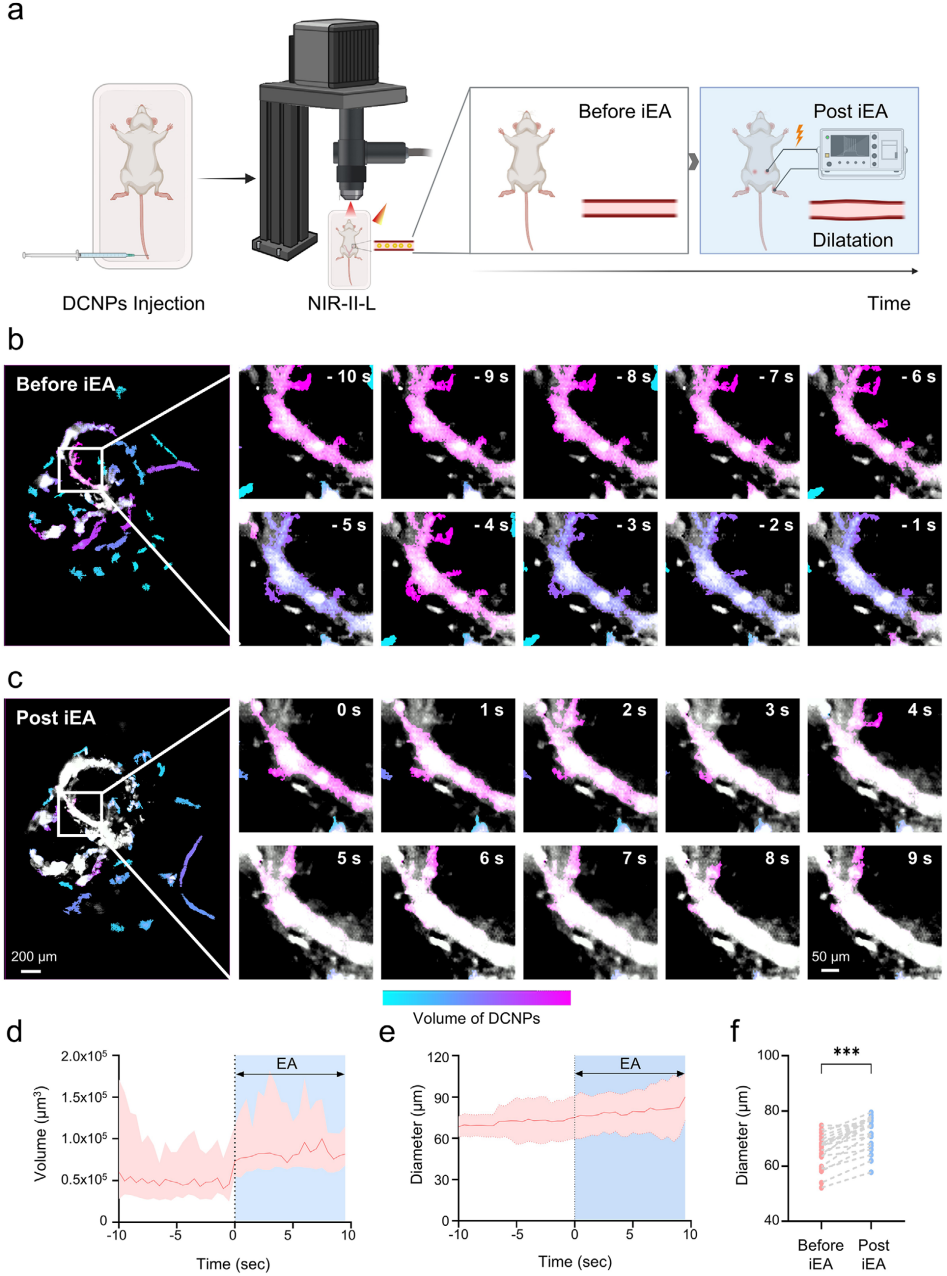
Real‐time NIR‐II‐L imaging and iEA‐induced transient vasodilation in the ovary. (a) Schematic of the experimental setup. Mice received intravenous injection of DCNPs for vascular labeling, followed by NIR‐II‐L imaging to visualize ovarian vasculature before and after immediate electroacupuncture (iEA). (b,c) Representative NIR‐II‐L time‐lapse images of ovarian vessels before (b) and after (c) iEA. Pseudo color scale (cyan to magenta) indicates relative volume of circulating DCNPs. Insets show magnified regions of interest at indicated time points (in seconds), demonstrating progressive signal enhancement and vessel dilation. (d) Time course of DCNPs signal volume in the entire ovary before and after the onset of iEA (*t* = 0 s). Data from a representative sham‐treated mouse are shown. Shaded area represents mean±95% confidence interval. A rapid rise in perfusion was observed within seconds following stimulation. (e) Time course of vessel diameter changes in a single ovarian arteriole before and after iEA onset (*t* = 0 s). Data are shown as mean±standard error of the mean (SEM). (f) Before‐after plots represent paired diameter values from 20 arterioles (3 mice per group) before and after iEA stimulation. Statistical comparisons were performed using the two‐sided Wilcoxon matched‐pairs signed rank test. *p < 0.05, **p < 0.01, ***p < 0.001; ns, not significant.

To assess the hemodynamic effects of a single immediate EA (iEA) session on ovarian microcirculation, we established a real‐time vascular imaging platform based on the NIR‐II region. Lanthanide nanocrystals, α‐NaYbF_4_:2%Er,2%Ce@NaYF_4_ with core–shell nanostructure were synthesized and used as high‐contrast bioimaging probes (Figure 1a,b and Figure S1). The fluorescent spectrum showed its emission peak located at around 1532 nm under 980 nm excitation (Figure 1c, red line). The fluorescent mechanism in Yb^3+^─Er^3+^─Ce^3+^ pair was a classical down‐shifting process that Ce^3+^ dopants could facilitate efficient non‐radiative phonon‐assisted cross relaxation of Er ^4^I_11/2_ state to the first excited ^4^I_13/2_ state, resulting in fluorescent enhancement at 1532 nm and suppressing its upconversion fluorescence (Figure 1d) [27, 28]. To reveal different examination performance in NIR region, we also provided the in vivo imaging comparison within different wavelengths. According to the photon‐tissue interaction theory, biological tissue absorption and scattering are the main factors that influenced fluorescent imaging contrast and resolution. In this regard, previous work has divided NIR‐II window into several sub‐regions, including NIR‐II‐L (1500‐1900 nm), NIR‐II short‐wavelength (NIR‐II‐S, 1000–1400 nm) and NIR‐II‐x (1400‐1500 nm) regions [29, 30]. Although water absorption could quench fluorescent intensity of imaging probes (Figure 1c, black line), it could also absorb scattering signals from biological tissues, thus enhancing the in vivo imaging resolution [31, 32]. Among these sub‐regions, NIR‐II‐L was proved to be a superior bioimaging window due to less photon scattering and minimized autofluorescence interference, while NIR‐II‐x usually relied on high brightness fluorescent probes. Here, in vivo bioimaging performance of ovarian vascular vessels was firstly compared within both NIR‐II‐L and NIR‐II‐S sub‐regions [33, 34]. Two lanthanide nanocrystals, including α‐NaYbF_4_:2%Er,2%Ce@NaYF_4_ emitted in NIR‐II‐L and α‐NaYbF_4_:10%Ho@NaYF_4_ emitted in NIR‐II‐S were both intravenous injection into the same mouse (Figure 1e). The vascular imaging performance of two distinct NIR‐II probes was separately compared before and post iEA. In vivo ovarian imaging showed that the NIR‐II‐L probe offered significantly better vascular delineation, as demonstrated by lower FWHM (Figure 1f). Time series images of NIR‐II‐L imaging confirmed that the probe predominantly circulated within the vasculature during the early post‐injection period, providing a stable imaging window for iEA experiments, followed by gradual hepatic accumulation and clearance at later time points (Figure S2). Thus, high‐resolution NIR‐II‐L imaging could precisely reflect several related vascular indicators, such as width, perfusion and fluorescence signals of vascular vessels. Following intravenous injection of α‐NaYbF_4_:2%Er,2%Ce@NaYF_4_ downconversion nanoparticles (DCNPs) [30], high‐fidelity fluorescence signals in the ovarian region could be continuously recorded (Figure 2a). EA was applied bilaterally at Sanyinjiao (SP 6) and Guilai (ST 29) using 2 Hz, 3 mA parameters under steady anesthesia. Image sequences were obtained before (‐10 to ‐1 s, before EA) and immediately after (0 to 9 s, post EA) stimulation onset.

Representative time‐series images (Figure 2b,c) were acquired to observe the hemodynamic response over a 20 s interval. During the baseline phase (before EA), the imaging intensity of DCNPs remained stable within the ovarian vasculature. The images displayed sharp vessel contours and minimal intensity fluctuation, indicating steady perfusion of blood vessels (Figure 2b). Upon initiation of EA, fluorescence intensity rapidly increased in parenchymal micro vessels, accompanied by a visible lateral dilation of the main supplying artery at the ovarian hilum (Figure 2c). These changes reflected a coordinated rise in flow velocity and perfusion volume, consistent with immediate vasodilation. Notably, such a response emerged within seconds of stimulation and was reproducible across mice, indicating a robust and temporally precise vascular activation induced by iEA. Further quantitative analysis (Figure 2d) showed that EA stimulation led to an immediate increase of approximately 65% in total DCNPs signal volume, indicating a rapid enhancement of ovarian microcirculatory perfusion. Figure 2e presents the diameter changes of a target vessel (the most prominent and morphologically stable artery located at the ovarian hilum) over time, demonstrating a maintained dilation during the stimulation period. Figure 2f summarizes the full width at half maximum (FWHM) analysis of this vessel across 20 arteries from 3 mice, which demonstrated an average diameter increase of approximately 11% after EA stimulation. Continuous NIR‐II‐L imaging over an extended time window showed that the iEA‐induced vasodilatory response was maintained during stimulation and reversibly resolved after stimulation cessation (Figure S3).

Notably, the immediate vasodilatory response exhibited a strong dependence on stimulation parameters. Parameter testing revealed that low‐frequency EA at 1 mA evoked significantly weaker responses compared to 3 mA (Figure S4a–c). Neither high‐frequency EA (100 Hz; Figure S4d–i) nor alternating frequency stimulation (2/100 Hz; Figure S5a–f) induced noticeable vasodilation. Moreover, stimulation at control acupoints did not alter ovarian perfusion (Figure S5g–i), supporting that EA‐induced vascular modulation is specific to particular parameters and acupoints. While manual acupuncture (MA) at specific acupoints could also elicit moderate increases in ovarian perfusion and vessel dilation, these effects were weaker compared to EA (Figure S6). Together, these results indicate that the immediate perfusion response is parameter‐dependent and acupoint‐specific, and we therefore adopted the 2 Hz, 3 mA setting as the primary iEA condition for subsequent mechanistic experiments.

### 2.2 iEA Alleviates Ovarian Hypo‐Perfusion in PCOS Models via Neurovascular Coupling

**FIGURE 3:**
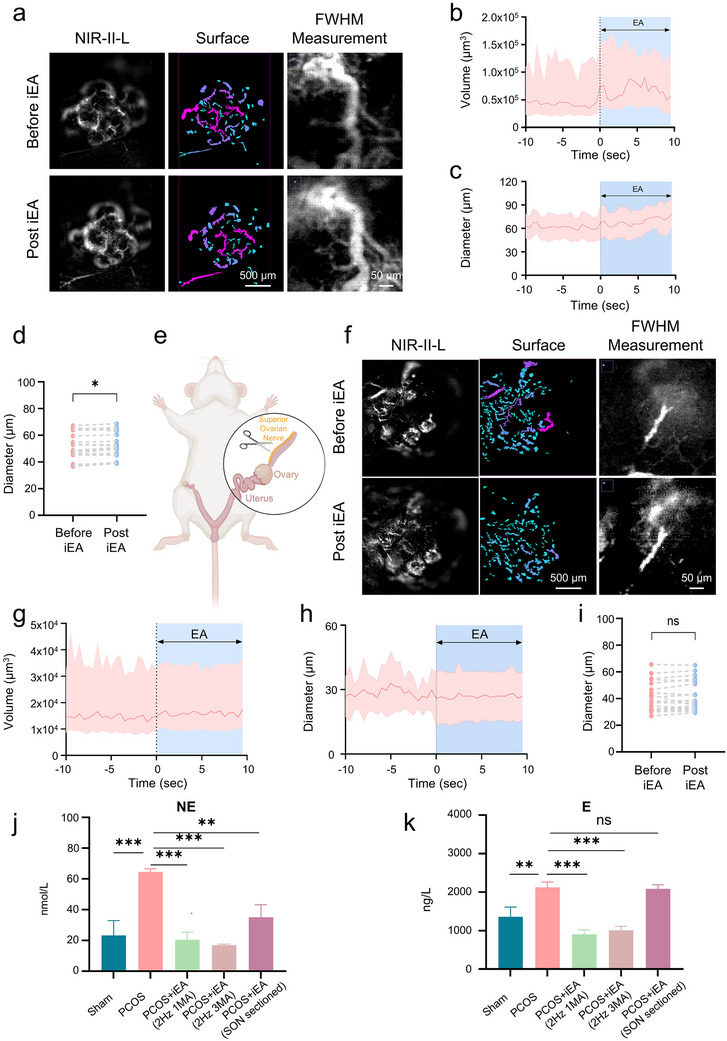
iEA‐induced vasodilation is impaired in PCOS mice and requires intact ovarian sympathetic innervation. (a) Representative NIR‐II‐L fluorescence, vessel surface reconstruction, and FWHM‐based diameter measurement in PCOS mice before and after iEA. b,c, Time course of DCNPs perfusion volume (b) and vessel diameter (c) in PCOS ovaries, aligned to EA onset at *t* = 0. Data shown as mean±95% CI (b) or mean±SEM (c). (d) Before‐after plots represent paired diameter values from 20 vessels (3 PCOS mice) before and after iEA stimulation. (e) Illustration of superior ovarian nerve transection procedure. (f) Representative NIR‐II‐L, vessel surface, and FWHM images before and after EA in SON sectioned treated PCOS mice. (g,h) Time course of DCNPs perfusion volume (g) and vessel diameter (h) in SON sectioned treated PCOS mice. Shown as mean ± 95% CI (g) or mean ± SEM (h). (i) Before‐after plots represent paired diameter values before and after iEA stimulation. (*n* = 20 vessels from 3 mice). (j) Ovarian NE levels across experimental groups (*n* = 6 per group). iEA significantly reduced NE levels in PCOS mice. (k) Ovarian E levels in each group (*n* = 6 per group). Statistical analysis was performed using one‐way ANOVA followed by Tukey's multiple comparisons test. *P* values were adjusted for multiplicity; *p < 0.05, **p < 0.01, ***p < 0.001. Before‐after plots were analyzed using the two‐sided Wilcoxon matched‐pairs signed rank test. *p < 0.05, **p < 0.01, ***p < 0.001; ns, not significant.

Impaired ovarian blood perfusion is a prevalent functional abnormality observed in PCOS, which has been associated with reduced vascular reactivity and microcirculatory dysregulation [35, 36]. To evaluate whether iEA can improve local blood flow in the ovaries of PCOS models, we employed NIR‐II‐L real‐time imaging to monitor dynamic vascular changes in a DHT‐induced PCOS mouse model. As shown in Figure 3a, iEA stimulation at Sanyinjiao (SP 6) and Guilai (ST 29) (2 Hz, 3 mA) led to a rapid increase in DCNPs fluorescence within ovarian vessels, indicating enhanced blood perfusion. Notably, lateral dilation of vessels was observed, and subsequent surface reconstruction combined with FWHM analysis confirmed vessel diameter expansion. Quantitative analysis revealed that the ovarian perfusion volume increased immediately following iEA stimulation by approximately 53.2% (Figure 3b,c). Further FWHM measurements across 20 vessels from 3 mice showed an average diameter increase of 8.6% during the post‐stimulation imaging window (Figure 3d), reflecting an immediate regulatory effect of iEA on ovarian microcirculation. It is noteworthy that the magnitude of this response was less pronounced compared to the sham group.

To further explore the mechanism underlying iEA‐mediated perfusion enhancement, particularly its dependence on sympathetic innervation, we surgically transected the Sympathetic ovarian nerve (SON) in PCOS mice (Figure 3e). The SON provides the principal sympathetic innervation to the ovary, extensively innervating the stromal and vascular compartments. Its activation is known to induce vasoconstriction and reduce perfusion [16, 37]. After SON transection, iEA stimulation failed to cause any significant change in perfusion volume or vessel diameter (Figure 3f–i), indicating that intact sympathetic innervation was essential for the vasodilatory effect of iEA. Consistently, ELISA showed elevated ovarian norepinephrine (NE) and epinephrine (E) levels in PCOS mice, both of which were significantly reduced following iEA (Figure 3j,k). Notably, these modulatory effects were largely abolished in mice with SON transection, further supporting that the observed neurochemical changes relied on neurovascular regulation mediated by sympathetic nerve.

Various EA protocols demonstrated differential attenuation of NE levels, with the most pronounced effects observed at 100 Hz (1 mA, 3 mA) and 2/100 Hz (1 mA). The reduction in E levels was more modest and only evident in specific conditions, without reaching statistical significance overall. Notably, control acupoint stimulation did not induce significant changes in either neurotransmitter, further highlighting the acupoint‐specific and parameter‐dependent nature of EA‐mediated effects (Figure S7a,b). Collectively, these findings demonstrated that iEA rapidly improved ovarian perfusion in PCOS through a vasodilation response that required intact neurovascular coupling. This effect was associated with suppression of NE release and attenuation of sympathetic over‐activity, which together contributed to the restoration of ovarian blood flow.

### 2.3 cEA Enhances Vascular Responsiveness and Follicular Development

**FIGURE 4:**
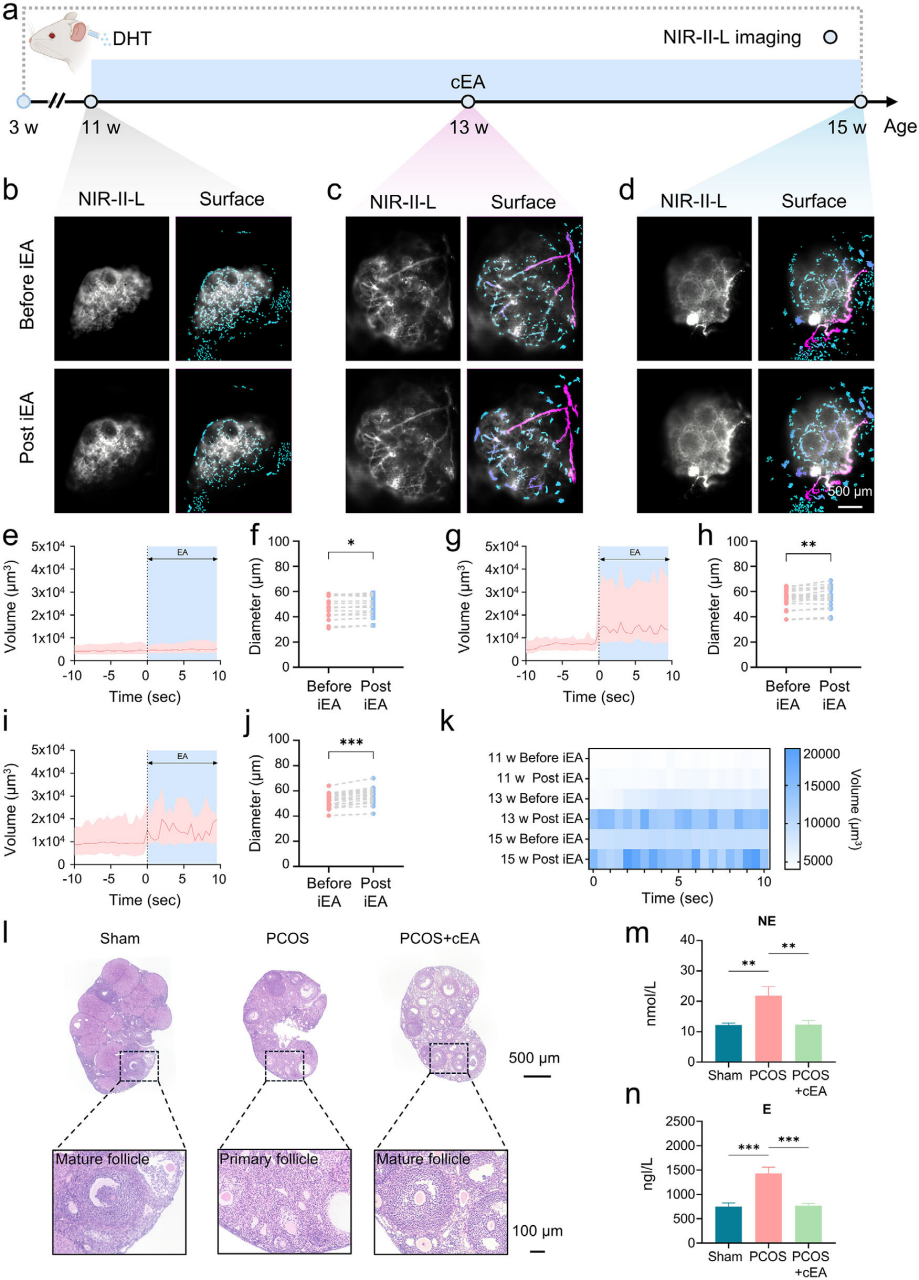
cEA progressively enhances ovarian neurovascular responsiveness and perfusion in PCOS mice. (a) Experimental timeline. DHT‐induced PCOS model mice received EA treatment starting at week 11. EA was administered once daily for five consecutive days per week, followed by two days of rest, for a total of four weeks. In vivo NIR‐II‐L imaging was performed at weeks 11, 13, and 15. (b–d) Representative NIR‐II‐L and surface‐rendered vascular images from the same PCOS mouse before and after iEA at week 11 (b), week 13 (c), and week 15 (d). e, Time course of ovarian perfusion volume before and after iEA at week 11, recorded in the same PCOS mouse shown in b. Data are plotted as mean±95% confidence interval. (f) Statistical comparison of ovarian vessel diameters before and after iEA stimulation at week 11 (*n* = 20 vessels from 3 PCOS mice). (g) Perfusion volume over time in the same mouse shown in c, at week 13. Data shown as mean±95% confidence interval, aligned to EA onset (*t* = 0). (h) Vessel diameter changes before and after iEA at week 13 (*n* = 20 vessels from 3 PCOS mice). (i) Perfusion volume over time in the same mouse shown in (d) at week 15. Data shown as mean±95% confidence interval, aligned to EA onset (*t* = 0). (j) Vessel diameter changes before and after iEA at week 15 (*n* = 20 vessels from 3 PCOS mice). (k) Heatmap of perfusion volume across imaging sessions at weeks 11, 13, and 15 in the same mouse shown in (e, g, i), aligned to EA onset (*t* = 0). (l) H&E staining of ovarian sections from Sham, PCOS, and PCOS+EA groups. (m,n) Ovarian levels of NE and E in Sham, PCOS, and PCOS+EA groups (*n* = 6 mice per group). One‐way ANOVA followed by Tukey's multiple comparisons test; *p < 0.05, **p < 0.01, **p < 0.001. Before‐after plots were analyzed using the two‐sided Wilcoxon matched‐pairs signed rank test. *p < 0.05, **p < 0.01, ***p < 0.001; ns, not significant.

To further evaluate the long‐term regulatory effects of EA on ovarian blood perfusion in PCOS, we employed a DHT‐induced mouse model and initiated periodic EA treatment (cumulative EA) from 11 weeks onwards (Figure 4a). Each treatment cycle consisted of five consecutive days of daily EA stimulation followed by 2 d of rest. This regimen was maintained for a total duration of 4 weeks. To assess whether this long‐term intervention enhanced the ovarian vascular response to EA, we performed standard single‐session EA stimulation (iEA) at three distinct time points including baseline (week 11), midpoint (week 13), and endpoint (week 15), and conducted in vivo NIR‐II‐L imaging to evaluate immediate blood flow changes. This design allowed us to monitor dynamic vascular reactivity following cEA. At baseline (week 11), ovarian vessels in PCOS mice appeared sparse and fragmented with visibly narrowed primary branches and discontinuities along the vascular tree, indicating the compromised perfusion and structural integrity. At this stage (Figure 4b), iEA induced only a mild increase in perfusion with minimal vessel dilation. Corresponding quantitative analysis revealed only a slight increase in perfusion volume and vessel diameter (Figure 4e,f), indicating a weak vascular response under these conditions. Conversely, at the mid (week 13; Figure 4c) and late (week 15; Figure 4d) phases of treatment, the same iEA stimulus evoked markedly greater vascular responses, including increased perfusion and maintained vessel dilation. Primary vessels exhibited more continuous morphology and recovered diameter, accompanied by improved clarity of microvascular branches (Figure 4g–j). These findings demonstrated that cEA not only sensitized ovarian vessels to neuromodulation but also promoted structural remodeling. For example, main trunk dilation and angiogenic reconstitution benefited from the restoration of local circulatory capacity. A time‐resolved heatmap further confirmed the progressive augmentation of perfusion response throughout treatment (Figure 4k). In contrast, PCOS mice that did not receive EA exhibited persistent impairment in perfusion and sparse vasculature across the same observation window (weeks 11–15, Figure S8), with no spontaneous recovery. Consistently, histological analysis revealed a paucity of mature follicles in untreated PCOS ovaries, while cEA restored follicular development, suggesting enhanced microenvironmental support for folliculogenesis (Figure 4l).

Neurotransmitter profiling further indicated that cEA alleviated sympathetic hyperactivity and modulated the ovarian microenvironment (Figure 4m,n). In the PCOS group, both NE and E levels were markedly elevated compared to the sham group. cEA significantly reduced NE concentrations, while E levels exhibited a decreasing trend under some stimulation parameters, though interindividual variability precluded statistical significance. These findings suggested that cEA restored sympathetic neurotransmitter balance in the ovary and mitigated neurogenic stress. Additional evidence demonstrated that cEA not only attenuated body weight gain and restored estrous cyclicity (Figure S9) but also markedly reversed hyperandrogenemia and gonadotropic axis disruption (Figure S10), thereby contributing to the systemic reprogramming of neuroendocrine dysregulation in PCOS. Compared to cEA, cumulative MA also showed modest improvements in hormone levels, body weight, and estrous cyclicity, and partially promoted structural contacts between neurovascular components and follicles (Figure S11).

Collectively, these results demonstrated that periodic EA stimulation (cEA) progressively enhanced neurovascular coupling in PCOS ovaries. By boosting local vascular responsiveness, reducing sympathetic tone, and promoting follicular maturation, EA exerted a multi‐layered therapeutic effect.

### 2.4 cEA Restores Follicular Development and Neurovascular Architecture in PCOS Ovaries

**FIGURE 5:**
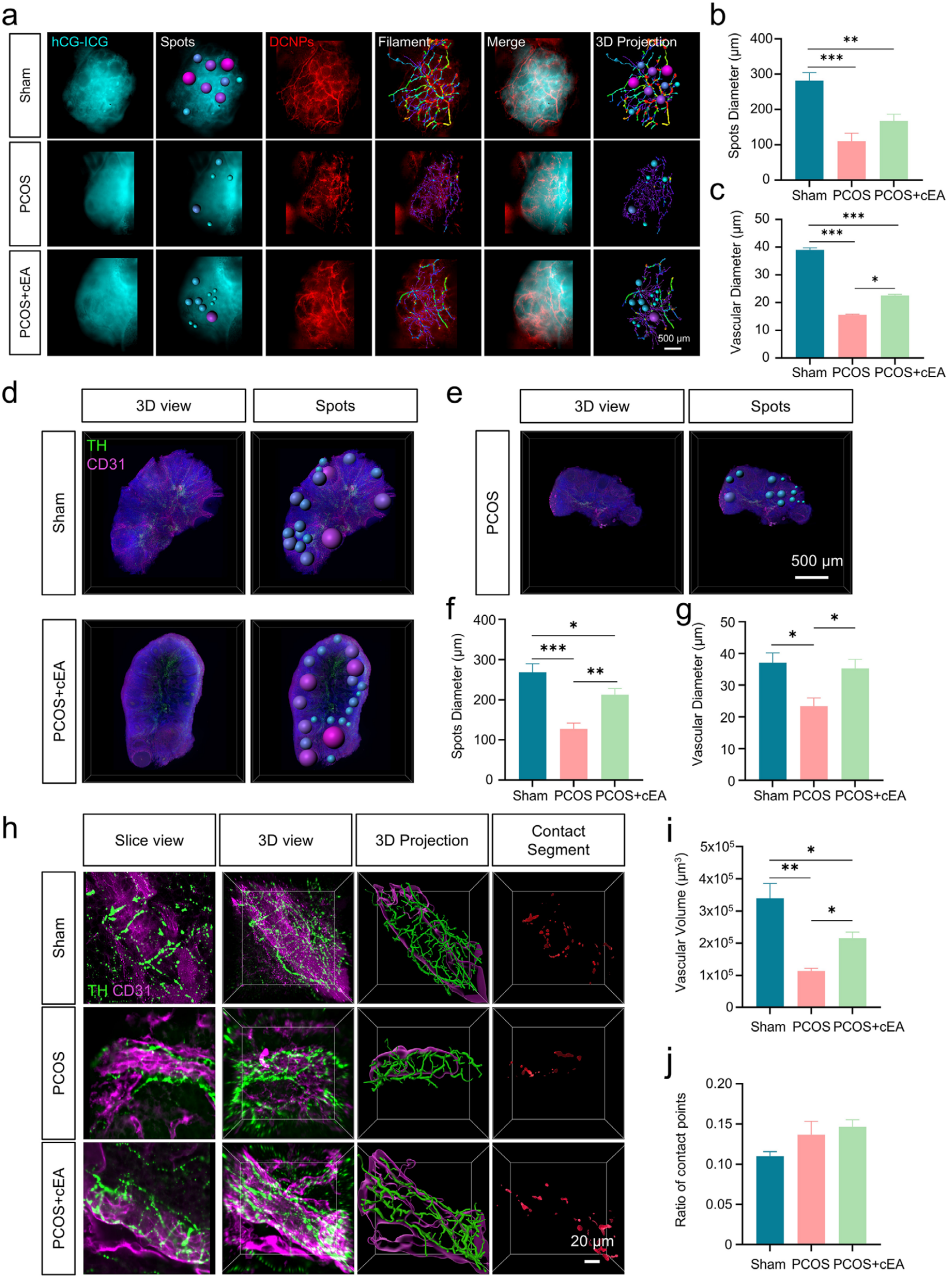
cEA remodels follicle–vasculature–nerve tracts and restores spatial neurovascular contact in PCOS ovaries. (a) Dual‐channel NIR‐II in vivo imaging of whole ovaries from Sham, PCOS, and PCOS+EA groups. Follicles were labeled with hCG‐ICG (cyan), and vasculature was visualized using DCNPs (red). Spots and filaments represent 3D reconstructions of follicles and vessels, respectively. (b,c) Quantification of follicle size (b) and average vascular diameter (c) across groups. (d,e) 3D imaging of whole ovaries stained for tyrosine hydroxylase (TH, green) and CD31 (magenta), showing sympathetic innervation and vasculature. Spots denote identified follicles. (f,g) Statistical comparisons of follicle diameter (f) and vascular diameter (g) from 3D‐immunostained ovaries. (h) High‐resolution 3D visualization of the neurovascular interface in ovarian sections. (i,j) Quantification of total vascular volume (i) and the ratio of nerve‐vessel contact points (j). Data are presented as mean±SEM; *n* = 3 mice per group. One‐way ANOVA followed by Tukey's multiple comparisons test; *p < 0.05, **p < 0.01, **p < 0.001.

To evaluate structural and functional abnormalities in folliculogenesis, vascular morphology, and sympathetic innervation in PCOS ovaries, and to examine the restorative potential of cEA, we implemented a multi‐channel imaging strategy combining in vivo NIR‐II fluorescence imaging [38] with ex vivo immunohistochemical reconstruction [12]. Based on our previously established multiplexed imaging protocols, as shown in Figure 5a, the sham ovaries exhibited multiple large follicles ranging from antral to preovulatory stages. hCG‐ICG probes specifically labeled the follicular walls to show their structural maturity, highlighting well‐formed fluid‐filled cavities and densely organized granulosa cell layers. DCNPs‐based vascular imaging revealed a radially organized capillary plexus tightly surrounding the follicular basement membrane, characteristic of robust perifollicular vascularization. By contrast, PCOS ovaries predominantly exhibited early‐stage or pre‐antral follicles with reduced diameters and indistinct antral formation. Corresponding vascular signals appeared fragmented and diminished, with narrowed capillaries and sparse branching (Figure 5b,c). cEA intervention markedly restored follicular and vascular architecture, increasing both the number and size of antral follicles. It also enhanced capillary continuity and circumferential alignment around follicle borders, suggesting synchronized improvements in folliculogenesis and microvascular support.

To validate in vivo observations, ex vivo 3D reconstruction of ovarian tissue stained for CD31 (vasculature) and tyrosine hydroxylase (TH; sympathetic axons) was performed (Figure 5d,e). Morphometric quantification showed reduced follicle count and CD31^+^ vascular density in PCOS, both of which were significantly restored after cEA treatment (Figure 5f,g), supporting the in vivo imaging findings. Neurovascular coupling constitutes a key anatomical foundation for stabilizing local blood perfusion and enabling fine‐tuned functional regulation. This coupling was particularly crucial in the ovary, which relies on highly dynamic microcirculatory perfusion. Moreover, sympathetic innervation directly modulates not only vascular tone but also potentially endocrine signaling [10, 39]. Analysis of neurovascular coupling revealed that TH^+^ nerves and CD31^+^ vessels were closely adjacent and densely distributed in the ovaries of the Sham group (Figure 5h). In PCOS, both the absolute number of TH–CD31 contact sites and the overall vascular volume were markedly reduced (Figure 5h,i), reflecting vascular rarefaction and diminished sympathetic innervation. When normalized to vascular volume, the contact ratio showed no significant difference between groups (Figure 5j), indicating preservation of proportional neurovascular coupling density despite vessel regression. cEA partially restored vascular volume and increased the absolute number of contacts, suggesting structural reinstatement of neurovascular integration. cEA exerted integrative effects on the ovarian microenvironment, supporting both follicular maturation and restoration of local hemodynamic homeostasis.

### 2.5 EA‐Induced Ovarian Vasodilation Requires Netrin‐1 Signaling

**FIGURE 6:**
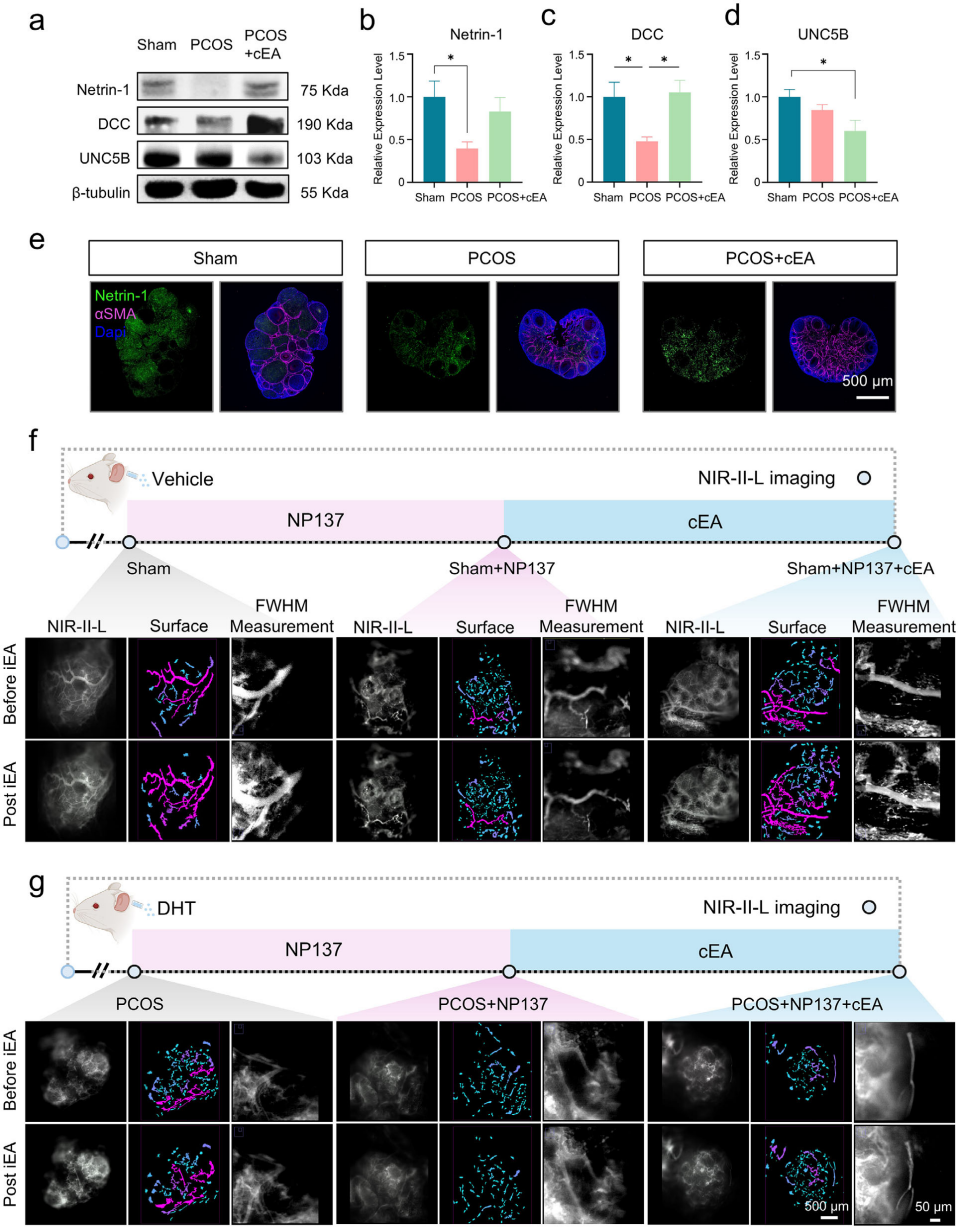
Netrin‐1 signaling mediates EA‐induced vasodilation in PCOS ovaries. (a) Western blot analysis of ovarian Netrin‐1, DCC, and UNC5B expression in Sham, PCOS, and PCOS+EA groups. (b–d) Quantification of protein band intensities from (a) normalized to β‐tubulin (*n* = 3 mice per group). One‐way ANOVA with Tukey's post hoc test. (e) Immunofluorescence staining of ovarian sections showing Netrin‐1 (green), α‐SMA (magenta), and nuclei (DAPI, blue) in Sham, PCOS, and PCOS+EA groups. (f) NIR‐II‐L imaging in Sham mice treated with or without NP137 (a Netrin‐1 neutralizing antibody), before and after iEA stimulation. DCNPs were used for vascular visualization, and vessel diameter was measured using FWHM analysis. (g) NIR‐II‐L imaging of PCOS, PCOS+NP137, and PCOS+NP137+EA groups before and after iEA. NP137 abolished EA‐induced vasodilation in PCOS ovaries. Data are shown as mean ± SEM. Statistical analysis was performed using one‐way ANOVA with Tukey's multiple comparisons test. *p < 0.05, **p < 0.01, **p < 0.001.

Previous studies have identified the axon guidance molecule Netrin‐1 as a key regulator not only of central nervous system development but also of peripheral neurovascular interactions, particularly in guiding sympathetic axons and establishing vascular innervation [40, 41]. To investigate whether this signaling pathway contributed to the vascular effects of EA in the ovary, we focused on Netrin‐1 and its canonical receptors DCC and UNC5B. We systematically examined their expression patterns and functional relevance across multiple intervention models. Western blot analysis revealed a significant reduction of ovarian Netrin‐1 protein expression in PCOS mice (Figure 6a–d), indicating impairment of this pathway under pathological conditions. After 4 weeks of cEA treatment, Netrin‐1 expression was notably restored, suggesting its pivotal role in mediating EA‐induced vascular regulation. Among its receptors, the chemo‐attractive receptor DCC exhibited a downward trend in PCOS and was upregulated after EA, whereas the repulsive receptor UNC5B showed no significant change. These findings implied that EA could facilitate neurovascular reconstruction by enhancing the pro‐guidance signaling of the Netrin‐1 axis. Immunofluorescence revealed broad Netrin‐1 expression and partial colocalization with α‐SMA^+^ vessels in sham ovaries. In PCOS, both signal intensity and vascular association were reduced, while cEA restored expression and vessel‐related clustering (Figure 6e), supporting its involvement in neurovascular coupling. During cEA treatment, ovarian Netrin‐1 expression exhibited a clear time‐dependent increase, with higher levels detected after 4 weeks of cEA compared with 2 weeks of treatment (Figure S12)

To assess the functional necessity of Netrin‐1 signaling, three treatment conditions were applied to both sham and PCOS mice: Vehicle control, local NP137 administration (a Netrin‐1 antagonist) [42], and NP137 combined with cEA. All groups received an iEA stimulus at week 15, followed by NIR‐II‐L imaging to evaluate vascular responses. The injection dose was determined based on the results of the pre‐experiment (Figure S13). In sham mice (Figure 6f), iEA elicited a robust increase in ovarian blood perfusion and vessel diameter, reflecting intact neurovascular responsiveness. However, blockade of Netrin‐1 with NP137 markedly attenuated both perfusion enhancement and FWHM expansion, indicating that even under physiological conditions, Netrin‐1 signaling contributed to the maintenance of vascular reactivity. Notably, when mice received 4 weeks of cEA following NP137 treatment, subsequent iEA stimulation restored vasodilation responses, suggesting that cEA could reestablish neurovascular integrity and partially compensate for the disrupted Netrin‐1 signaling (Figure S14a–f). In the PCOS context (Figure 6g), baseline vascular responsiveness was significantly impaired. However, in the presence of NP137, even after full cEA intervention, iEA failed to induce effective vascular responses, and basal perfusion levels remained low. This suggested that blockade of Netrin‐1 disrupted the neurovascular remodeling process critical for EA‐mediated functional recovery in PCOS ovaries (Figure S14g–l).

Together, these results identify Netrin‐1 as a key regulator of ovarian vascular reactivity under both physiological and pathological conditions. Its expression is essential for the neurovascular remodeling and perfusion enhancement elicited by EA, reflecting a fundamental structural and functional dependency of EA effects on Netrin‐1 integrity.

### 2.6 Inhibition of Netrin‐1 Causes Neurovascular Uncoupling and Ovarian Vascular Defects

**FIGURE 7:**
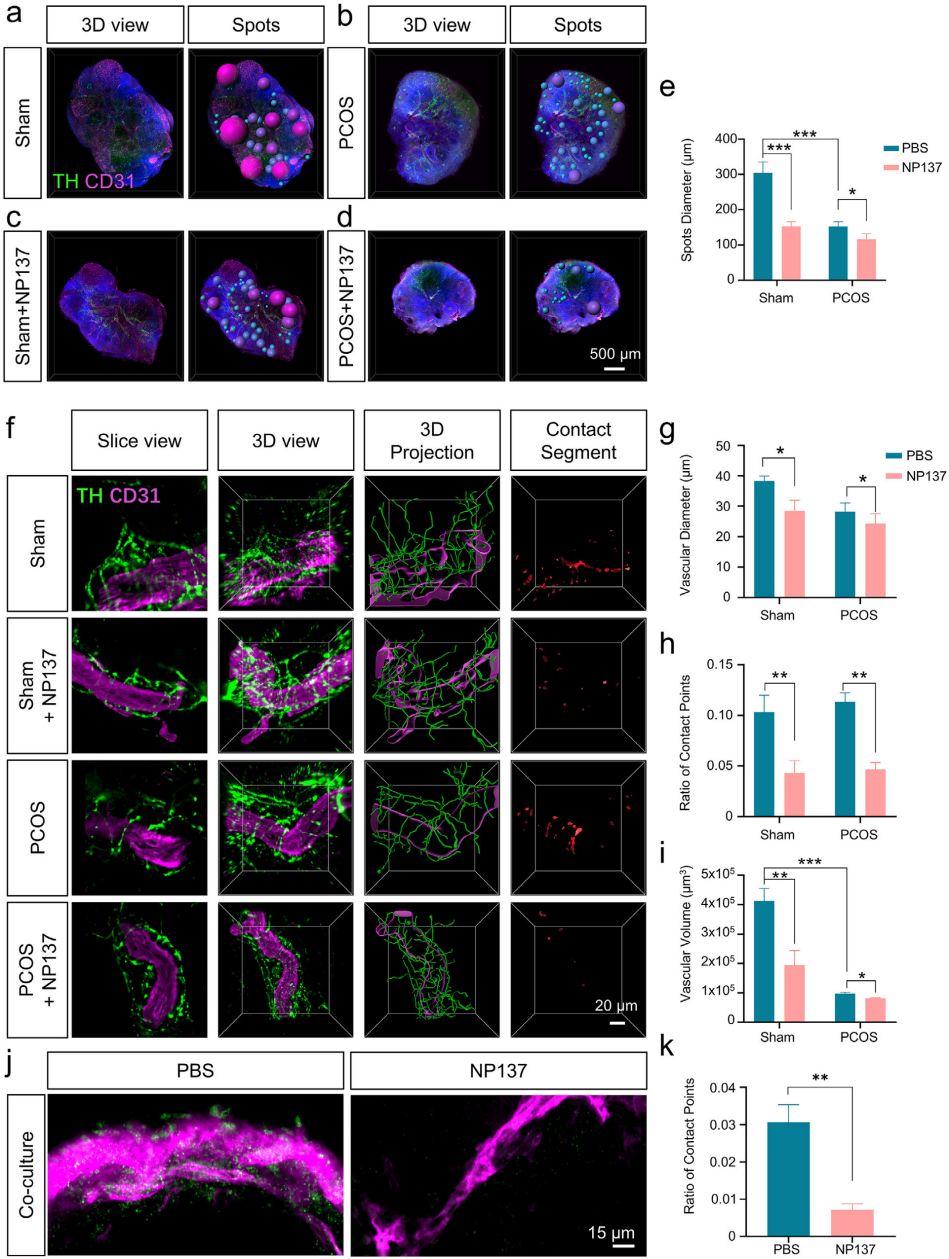
Netrin‐1 blockade disrupts neurovascular coupling and impairs follicular development in vivo and in vitro. (a–d) 3D rendering of whole ovaries from Sham, PCOS, Sham+ NP137 and PCOS+NP137 group mice. (e) Quantification of follicle diameter across groups (*n* = 3 mice). (f) High‐resolution 3D imaging of TH‐CD31 contact in ovarian slices. (g–i) Quantification of vascular diameter (g), proportion of contact points (h), and vascular volume (i) in Sham and PCOS ovaries treated with or without NP137. (j) Confocal images of sympathetic axon‐endothelial contact in TH/CD31‐labeled co‐cultures (treated with PBS or NP137). (k) Quantification of contact point ratio in (j) (*n* = 3). Data are presented as mean±SEM. Two‐way ANOVA followed by Sidak's multiple comparisons test; Panel (k) was analyzed using an unpaired two‐tailed Student’s t test. *p < 0.05, **p < 0.01, **p < 0.001.

To examine whether Netrin‐1 plays a structural role in neurovascular architecture, we investigated its role in maintaining sympathetic‐vascular connectivity. Previous reports have shown its sustained upregulation during cEA alongside restored perfusion responsiveness. 3D imaging and in vitro validation were conducted to clarify its function in ovarian neurovascular coupling. Following local NP137 administration in both sham and PCOS ovaries, 3D reconstructions revealed substantial morphological alterations. In sham controls, follicles maintained intact architecture with dense CD31^+^ vasculature. NP137 treatment significantly reduced follicle volume and further exacerbated follicular underdevelopment in PCOS mice, implicating that Netrin‐1 supports follicular structural integrity (Figure 7a–e). Notably, NP137 alone did not affect body weight gain but markedly disrupted the estrous cycle rhythm. EA successfully corrected this disruption in non‐PCOS mice, whereas its regulatory effect was markedly diminished in the PCOS background under NP137 administration (Figure S15). 3D spatial mapping of TH^+^ fibers and CD31^+^ vessels (Figure 7f) revealed extensive neurovascular co‐alignment in sham controls. NP137 disrupted this organization, reducing contact density and structural parallelism. PCOS mice exhibited baseline coupling deficits, which were further worsened by NP137. Quantification confirmed smaller vessel diameters and fewer neurovascular contact points (Figure 7g,h). Total CD31^+^ vascular volume was significantly reduced following NP137 treatment (Figure 7i), supporting an essential role for Netrin‐1 in vascular maintenance and microvascular stability. To assess whether the disruption of neurovascular coupling involved direct structural interactions, we established an ex vivo co‐culture model of superior cervical ganglia (SCG) and isolated abdominal aorta segments, and administered NP137 during the co‐culture period (Figure 7j and Figure S16). Confocal imaging revealed that NP137 significantly decreased the number of close contacts between TH^+^ sympathetic terminals and the vascular endothelium (Figure 7k), implying that Netrin‐1 may contribute to the structural interface between sympathetic axons and vessels.

Collectively, these findings demonstrate that Netrin‐1 not only governs functional vascular responses but also maintains the structural interface between sympathetic axons and blood vessels. Its inhibition leads to neurovascular uncoupling, vascular regression, and impaired follicular development. These features align with PCOS‐associated perfusion deficits and highlight Netrin‐1 as a key node in EA‐mediated vascular restoration.

## 3 Discussion

This study showed that EA exerts dose‐ and time‐dependent neuromodulatory effects on ovarian perfusion in a PCOS model, encompassing both rapid vasomotor activation and progressive structural remodeling. To meet the demand for visualizing perfusion dynamics in deep organs, we established a real‐time imaging platform based on high‐resolution NIR‐II‐L fluorescence. Two NIR‐II lanthanide nanoprobes, including Ho‐based (emitted in NIR‐II‐S) and Er‐based (emitted in NIR‐II‐L) lanthanide nanocrystals, were firstly compared in ovarian vascular vessels. In vivo bioimaging performance shows that NIR‐II‐L region can offer superior images that truly reflect the vessels’ structural changes before and after EA. We demonstrate that iEA rapidly induces ovarian vasodilation consistent with sympathetic neurovascular reflexes, while cEA gradually reconstructs the neurovascular interface, restoring the spatial coupling between sympathetic terminals and blood vessels. Notably, these processes are partially dependent on the Netrin‐1 signaling pathway, which governs the spatial alignment and guidance of sympathetic axons with endothelial structures. Overall, our findings reveal that EA not only modulates functional outputs but also alters neurovascular architecture in a dose‐responsive manner, thereby reinstating ovarian homeostasis through structural intervention and providing a mechanistic rationale for non‐hormonal therapy in PCOS.

EA activates somatosensory afferents with excitability determined by parameters such as intensity, frequency, and needle depth [43, 44]. Electrophysiological studies have demonstrated that low‐intensity stimulation preferentially recruits large‐diameter, myelinated Aβ and Aδ fibers, whereas higher intensities are required to activate unmyelinated C fibers [45]. Afferent signals ascend via segmental and supraspinal pathways to engage central autonomic circuits, forming somato‐autonomic reflex arcs that modulate sympathetic outflow [46]. Previous studies have demonstrated that EA modulates gastrointestinal motility, cardiovascular tone, and ovarian perfusion through such neural pathways [47, 48, 49]. Once activated, sympathetic efferents provide rapid and spatially precise regulation over visceral blood flow. Sympathetic outputs from the ovarian plexus modulate local blood flow and steroidogenesis through NE signaling via α‐adrenergic receptors [50]. Notably, these axonal terminals release NE predominantly in a non‐synaptic fashion, with neurotransmitter diffusion into the extracellular space targeting adjacent vascular smooth muscle cells [51]. The differential responses NE and E likely reflect their distinct sources and regulatory mechanisms. NE is predominantly released from local sympathetic nerve terminals within the ovary and directly reflects regional sympathetic tone, whereas E is mainly secreted by the adrenal medulla and reaches the ovary via systemic circulation [52]. Accordingly, EA may preferentially attenuate local NE signaling, while modulation of circulating E could be more parameter‐sensitive and less consistent across stimulation protocols. Collectively, these findings delineate a well‐defined somatosensory‐sympathetic‐vascular regulatory axis, through which EA exerts functional control over ovarian blood flow, offering both anatomical and mechanistic rationale for its therapeutic utility in reproductive disorders.

Conventional in vivo imaging techniques for blood flow suffer from notable limitations, especially when applied to deep‐seated organs such as the ovary. Laser Doppler flowmetry (LDF) is restricted to superficial microcirculation and is highly susceptible to artifacts from tissue optical properties and motion [53]. Respiratory motion further complicates the localization of the ovary and the tracking of perfusion. These often provide only regional perfusion indices or trends, without resolving vascular architecture or capturing real‐time changes induced by interventions such as EA. Recent advances in high‐resolution NIR‐II‐L window (1500–1900 nm) have addressed these shortcomings [54, 55]. Lanthanide nanocrystals with abundant long‐wavelength emission could provide superior imaging performance with minimized tissue scattering and autofluorescence. While earlier studies primarily focused on structural imaging or probe development, few have explored real‐time physiological regulation or therapeutic response. Here, the established functional imaging paradigm based on NIR‐II‐L‐emitted DCNPs can capture both vascular architecture and perfusion dynamics, enabling dynamic quantification of EA‐induced neurovascular modulation. NIR‐II‐L imaging introduces a transformative approach for investigating neurovascular regulation in deep, highly perfused organs. The ovary, with its cyclical endocrine activity and coordinated neurovascular architecture, represents a unique system in this regard [56]. However, technical barriers have historically limited the in vivo exploration of ovary‐specific neurovascular units (NVUs) [10, 57]. NIR‐II‐L imaging now allows high‐resolution, dynamic visualization of ovarian blood flow and vessel organization, providing a novel framework for dissecting neurovascular coupling under physiological and pathological conditions.

In contrast to the inflammation‐sensing NVUs in the liver [58] or the motility‐regulating NVUs in the intestine [59], the ovarian NVU exhibits a unique triad of neurovascular‐endocrine integration, serving the reproductive functions of follicular development and hormone secretion. Sympathetic nerve fibers predominantly enter the ovary via vascular routes within the ovarian hilum and terminate around preantral to antral follicles, where they regulate local blood flow and steroidogenesis, forming a specialized neurovascular‐follicular unit [37, 60]. Structurally, the ovarian NVU is characterized by the parallel alignment of sympathetic axons with vasculature, enabling precise neurovascular contact at the follicular interface [61]. Functionally, it is highly responsive to both endocrine milieu and external stimuli, displaying a degree of plasticity and intervention sensitivity not commonly observed in other peripheral NVUs [62]. This functional coupling allows the ovarian NVU to rapidly adapt to internal physiological changes (e.g., fluctuating hormone levels) or external interventions such as EA. During each ovulatory cycle, the ovary undergoes cyclical waves of neurovascular remodeling, highlighting its exceptional dynamism and structural plasticity. Such plasticity underlies the NVU's capacity to facilitate follicle maturation and maintain endocrine homeostasis. The neurovascular‐follicular synergy, therefore, forms a critical physiological foundation for reproductive health [10].

Notably, the coupling integrity of the ovarian NVU can be disrupted under pathological conditions. In PCOS, despite an overall increase in sympathetic tone, local innervation around developing follicles is markedly diminished, particularly within the ovarian hilum and dominant follicle regions, a phenomenon often described as neuronal dropout [10]. Concurrently, vascular architecture becomes abnormally dilated and fragmented, indicating a breakdown of the coordinated neurovascular‐follicular interface. Such structural decoupling may contribute to impaired steroidogenesis, dysregulated perfusion, and subsequent follicular atresia. As previously proposed, alterations in ovarian innervation patterns are not merely epiphenomena but may actively participate in the pathogenesis and progression of PCOS [63]. The selective loss of neurovascular integration at critical follicular sites underscores the importance of spatially organized sympathetic signaling in maintaining ovarian function and highlights the NVU as a potential target for therapeutic intervention.

Strikingly, the physiological effects of EA display temporal specificity, with distinct mechanisms engaged at various time scales. iEA rapidly activates preexisting neurovascular pathways, leading to transient ovarian vasodilation and enhanced perfusion via classical somato‐sympathetic reflex arcs. In contrast, repeated stimulation through cEA gradually induces structural remodeling within the ovarian microenvironment, including sympathetic axon anchoring, vascular patterning, and reinforcement of neurovascular pairing. Our NIR‐II‐L imaging and 3D reconstructions revealed that cEA significantly enhances neurovascular proximity and restores the disrupted neurovascular‐follicular triad in the PCOS model. Mechanistically, this structural reorganization appears to be critically dependent on the Netrin‐1 signaling pathway. Previous studies have established Netrin‐1 as a key guidance cue coordinating sympathetic innervation and vascular patterning and have shown that its expression and release can be dynamically regulated by neuronal activity and electrical stimulation [64, 65, 66, 67]. Building on this evidence, our findings support a model in which cEA engages neurovascular plasticity mediated by Netrin‐1 to drive structural remodeling of the ovarian neurovascular unit and enhance responsiveness to iEA. In this study, blockade of Netrin‐1 with the neutralizing antibody NP137 markedly attenuated the neurovascular restoration induced by cEA and impaired follicular development, underscoring the essential role of this pathway in EA‐driven structural remodeling. Together, these findings support a regulatory paradigm wherein short‐term EA elicits immediate perfusion responses via reflex circuits. At the same time, long‐term EA promotes neurovascular reconstruction, thereby reestablishing tissue homeostasis at the structural level. The structural integrity of the ovarian NVU not only influences local perfusion and follicular fate but may also serve as a predictor of therapeutic responsiveness to EA. Using advanced imaging modalities such as NIR‐II‐L fluorescence, real‐time assessment of neurovascular architecture could enable dual‐dimensional patient stratification. Based on both structure and function, it helped identify PCOS subtypes with perfusion deficits or neurovascular decoupling. For patients with poor tolerance or limited response to hormonal therapy, EA could provide a non‐hormonal, reversible, and plastic route to intervention, activating somato‐sympathetic reflexes and restoring NVU integrity. Integrating NIR‐II‐L imaging with EA may yield precision strategies for a broader range of reproductive disorders.

Despite demonstrating that EA improves the ovarian microenvironment in PCOS via Netrin‐1‐mediated neurovascular remodeling, several questions remain unanswered. The source and receptor expression of Netrin‐1 in the ovary need clarification. Additionally, the expression patterns of its canonical receptors, such as DCC and UNC5B, have not yet been systematically characterized in ovarian tissue. Further studies are required to assess endocrine endpoints and the role of parasympathetic regulation.

## 4 Conclusion

In summary, our study uncovers a previously underexplored dose‐response mechanism through which EA restores ovarian function in PCOS via real‐time modulation of neurovascular coupling. By integrating lanthanide downconversion nanocrystals emitted in high‐resolution NIR‐II‐L imaging with volumetric immunoarchitecture reconstruction, we demonstrate that EA not only induces immediate vasodilatory responses via Netrin‐1‐dependent sympathetic‐endothelial signaling but also promotes long‐term reorganization of neurovascular‐follicular networks. These findings identify the ovarian neurovascular unit as a structurally and functionally responsive target of EA, and suggest that modulating neurovascular alignment may represent a novel strategy for reprogramming the dysfunctional ovarian microenvironment in PCOS.

## Author Contributions

Y.F., Y.W., and Z.C. conceived the experiments, designed the project and protocols, and developed the collaborations. Y.W., Z.C., J.L., W.H., Y.C., Y.W., X.T., W.L., and Y.Y. performed the experiments. Y.W. and Y.X. analyzed the results. Y.W., Y.F., and Z.C. wrote the manuscript. F.Z. and Y.F. provided scientific oversight and guidance and edited the manuscript. Y.W., F.Z., and Y.F. are the guarantors of this work and, as such, had full access to all of the data in the study and take responsibility for the integrity of the data and the accuracy of the data analysis. All authors read and approved the final manuscript.

## Funding

This work was supported by the National Natural Science Foundation of China (NSFC 81973945 and 82174497 to Y.F., NSFC 82505741 to W.H.), the Development Project of Shanghai Peak Disciplines‐Integrated Chinese and Western Medicine (20180101 to Y.F.), the China Postdoctoral Science Foundation (2025M780913 to Z.C.) and the Innovative Research Team of High‐Level Local Universities in Shanghai.

## Conflicts of Interest

The authors declare no conflicts of interest.

## Supporting information




**Supporting File 1**: advs74116‐sup‐0001‐SuppMat.pdf.

**Supporting File 2**: advs74116‐sup‐0002‐VideoS1.mp4.

## Data Availability

The datasets used or analyzed during the current study are available from the corresponding author on reasonable request.
