# Supplementary material for: Real‐Time and High‐Resolution NIR‐II‐L Imaging of Netrin‐1‐Mediated Neurovascular Coupling Driven by Dose‐Dependent Electroacupuncture
Source: Adv Sci (Weinh). 2026 Jan 27;13(19):e20847. doi: 10.1002/advs.202520847 (PMC13045393; doi:10.1002/advs.202520847)
Supplement: Supplementary file 1 — Supporting File 1: advs74116‐sup‐0001‐SuppMat.pdf. [file ADVS-13-e20847-s002.pdf]

Supporting Information

Real-Time and High-Resolution NIR-II-L Imaging of Netrin-1-Mediated Neurovascular Coupling Driven by Dose-Dependent Electroacupuncture

*Yicong Wang, Zi-Han Chen, Jiajia Li, Wei Hu, Ying Cao, Yu Wang, Xiaoyu Tong, Wenhan Lu, Yuze Yang, Yuehao Wang, Yan Xiao, Wenhao Gao, Yuning Chen, Yuyan Hou, Fan Zhang*and Yi Feng**

Materials and Methods

Animals and Ethics

All animal procedures in this study were conducted in accordance with the Criteria of the Medical Laboratory Animal Administrative Committee of Shanghai and were approved by the Ethics Committee for Experimental Research of Shanghai Medical College, Fudan University (Approval No. 20190221-079). Female BALB/c mice aged 21 days were obtained from Slack Laboratory Animal Co., Ltd. (Shanghai, China). Mice were housed in the SPF facility at Fudan University under a controlled 12-hour light/dark cycle with ad libitum access to food and water.

Synthesis of α-$NaYbF_4$:2%Er,2%Ce@$NaYF_4$ lanthanide downconversion nanocrystals

The synthesis method of α-$NaYbF_4$:2%Er,2%Ce@$NaYF_4$ lanthanide nanocrystals was referred to our previous report[1]. In brief, 1 mmol CF_3COONa, 0.96 mmol $Yb(CF_3COO)_3$, 0.02 mmol $Er(CF_3COO)_3$ and 0.02 mmol $Ce(CF_3COO)_3$ were added to a mixture of oleic acid (10 mmol), 1-octadecene (20 mmol) and oleylamine (10 mmol) in a three-necked flask at room temperature. The solution was then heated to 100 ° C for 30 min under vacuum and vigorous magnetic stirring to remove residual water. Next, the solution was heated to 310 ° C in 14 min under argon gas protection. The solution was maintained at 310 ° C for 40 minutes. After cooling to room temperature, the synthesized α-$NaYbF_4$:2%Er,2%Ce core nanocrystals were centrifuged and washed three times with ethanol and finally dispersed in 10 mL cyclohexane for further use.

Using the as synthesized core nanocrystals as seeds, α-$NaYbF_4$:2%Er,2%Ce@$NaYF_4$ core-shell nanostructure could be gained by the same epitaxial growth method. In details, 0.2 mmol seeds (dispersed in 2 mL cyclohexane), 0.6 mmol CF_3COONa (81.6 mg) and 0.6 mmol $Y(CF_3COO)_3$ (256.8 mg) were used as the precursors for epitaxial growth of $NaYF_4$ shell. The above seeds and precursors were firstly mixed with 3.2 mL oleic acid and 4.8 mL 1-octadecene in a three-neck 50-mL round-bottom flask with Schlenk line and a thermocouple temperature sensor. Then the solution was heated to 100° C to remove cyclohexane, residual water and methanol under vaccum. Afterward, the clear solution was heated to 290 ° C for 60 min under a gentle Ar flow. When the reaction system was cooled down to room temperature, the synthesized nanocrystals were centrifuged and washed three times with ethanol and finally dispersed in 2 mL cyclohexane for further use.

PCOS Model Establishment

To induce PCOS-like phenotypes, 3-week-old female mice were implanted subcutaneously in the dorsal neck region with DHT-releasing tubes (Cat# A8380, Sigma-Aldrich), each containing ~15 mg dihydrotestosterone (DHT) for sustained release over 12 weeks. This model recapitulates major features of human PCOS, including hyperandrogenism, anovulation, and metabolic disturbances. Control mice received empty tubes without DHT. Animals were randomly assigned to groups using a computer-generated randomization sequence, with body weight balanced across groups.

After 6 weeks of DHT exposure, estrous cycle stages were evaluated daily via microscopic examination of vaginal smears. The stages were identified based on dominant cell types: proestrus (predominantly nucleated epithelial cells), estrus (mainly cornified, anucleated cells), metestrus (a mixture of leukocytes, cornified cells, and nucleated epithelial cells), and diestrus (primarily leukocytes with occasional epithelial cells).

Live NIR-II-L Vascular Imaging

Mice were anesthetized with isoflurane and placed on a thermostatic imaging stage. A total of 100 μL of DCNPs ($NaYbF_4$:2%Er,2%Ce@$NaYF_4$) was injected via the tail vein. NIR-II fluorescence imaging was performed using an *in vivo* imaging system (Shanghai United Digital Biotech. Co. Ltd., NIR-II-ST, China), equipped with a thermoelectrically cooled InGaAs camera (NIRvana 640, Teledyne Princeton Instruments, 640×512 pixels), a 50 mm SWIR lens, and 980 nm laser excitation. A 1500 nm long-pass filter was used, with an exposure time of 500 ms. Image acquisition was conducted at two key time points: first, immediately after contrast stabilization in the vasculature, and second, during the EA stimulation. A stereo zoom microscope (Gemini, Artemis) with a fixed 1× objective and a 0.64×-4.50× zoom lens was used to capture wide-field vascular images.

To compare the spatial resolution between different NIR-II sub-regions, we conducted *in vivo* imaging using the NIR-II-S fluorescent probe. For NIR-II-S imaging, Ho-1152 dye was injected under the same conditions, and fluorescence was collected using a 1150 nm long-pass filter. All imaging was performed using identical camera settings and magnification parameters. Full width at half maximum (FWHM) and signal-to-noise ratio (SNR) were calculated by extracting cross-sectional intensity profiles from selected vascular regions.

EA Intervention

Immediate EA

iEA was performed under isoflurane anesthesia using an EA device (HANS LH202, Huawei Co., Beijing, China). Stainless steel acupuncture needles (Φ0.22 × 13 mm) were inserted bilaterally at designated acupoints and connected to the device. Three stimulation patterns were applied in separate groups: low-frequency (2 Hz), high-frequency (100 Hz), and alternating frequency (2/100 Hz). The current intensities were 3 mA and 1 mA, respectively, and the continuous stimulation time was 5 minutes.

The treatment group received EA at bilateral Sanyinjiao (SP6) and Guilai (ST29). Based on mouse anatomical landmarks, ST29 was localized approximately 3.5 mm lateral to the midline and 6.5 mm inferior to the umbilicus on the lower abdomen. SP6 was located approximately 5 mm above the medial malleolus on the posterior limb.

For the control group, acupuncture was applied at non-specific sites: bilateral Shenshu (BL23, 3 mm lateral to the second lumbar vertebra) and Neiguan (PC6, 2 mm proximal to the wrist crease on the inner forelimb).

Cumulative EA

cEA was initiated from the 8th week after PCOS model induction. Mice received low-frequency EA (2 Hz, 3 mA) for 30 minutes daily, five days per week, followed by a two-day rest, for four consecutive weeks. The stimulation sites for cEA were identical to those used in iEA: bilateral ST29 and SP6.

Bilateral SON Transection

Under isoflurane anesthesia, dorsolateral incisions were made at the midpoint between the costal margin and the anterior superior iliac spine, along the lateral abdominal wall. The incision involved the skin, abdominal musculature, and peritoneum, with care taken to avoid injury to internal organs. Through the incisions, both ovaries were gently exteriorized. Under a stereomicroscope, the ovarian suspensory ligament was carefully isolated using fine forceps, and the SON within the ligament was transected, as indicated in the schematic illustration (Fig. 2e). To ensure procedural consistency, the right SON was always severed before the left[2].

NP137 Intervention

At 8 weeks of age, both sham and PCOS mice were anesthetized with isoflurane and subjected to dorsolateral incisions, approximately 1 cm below the last rib on each side, as described in the SON transection procedure. Under a stereo dissecting microscope, both ovaries were gently exteriorized. NP137 (Cat# DHB70501, Antibody System, USA) was injected subcapsularly into each ovary using a microsyringe equipped with a pulled-glass capillary (10 µL capacity). The dosage was 2.5 µL per side (1.3 mg/mL), equivalent to 3.25 µg per ovary and 6.5 µg per mouse in total. In the control group, an equal volume of sterile PBS was injected at the exact anatomical location using the same protocol. After injection, the ovaries were returned to the abdominal cavity, and the muscle and skin layers were sutured separately to ensure closure.

Western Blotting

Total protein was extracted from mouse ovaries using RIPA lysis buffer containing protease and phosphatase inhibitors (Cat# P0013B, Beyotime) in a pre-chilled tissue homogenizer. Samples were incubated on ice for 30 minutes and centrifuged at 12,000 × g for 15 minutes at 4°C to collect the supernatant. Protein concentrations were normalized, and equal amounts of denatured lysates were separated by SDS-PAGE and transferred to PVDF membranes. After blocking in 5% non-fat milk, membranes were incubated overnight at 4°C with primary antibodies against Netrin-1 (1:1000, ab126729, Abcam), DCC (1:1000, PA547951, Thermo), UNC5b (1:1000, ab313565, Abcam), and β-Tubulin (1:5000, 10094-1-AP, Proteintech). After TBST washes, membranes were incubated with HRP-conjugated secondary antibodies at room temperature for 2 hours. Protein bands were visualized using enhanced chemiluminescence (ECL, WBKLS0500, Millipore), imaged with the ImageQuant LAS4000 system (GE Healthcare), and quantified using ImageJ.

Tissue Clearing

Ovarian tissues were cleared using a modified CUBIC protocol[3]. Fixed ovaries were immersed in delipidation solution S1 (150 g urea, 100 g N-butyldiethanolamine, 100 g Triton X-100 dissolved in 650 g ddH_2O) at 37 °C for 7 days with solution replacement every two days. After immunofluorescence staining, tissues were immersed in RI matching solution S2 (250 g urea, 225 g sucrose, 225 g antipyrine dissolved in 200 g ddH_2O and mixed with 100 g triethanolamine at room temperature after cooling) for refractive index matching. 3D imaging was performed using a BC43 benchtop confocal microscope (Andor Technology, Oxford Instruments, UK).

Immunofluorescence Staining

Ovarian tissues were fixed in 4% paraformaldehyde, dehydrated, and embedded in paraffin. Sections were dewaxed, rehydrated, and subjected to antigen retrieval, permeabilization, and blocking. Primary antibodies including anti-TH (1:500, ab112, Abcam), anti-CD31 (1:250, AF3628, R&D Systems), anti-Netrin-1 (1:250, AF1109, R&D Systems), and anti-α-SMA (1:1000, ab7817, Abcam) were applied overnight at 4°C. After PBS washes, sections were incubated with fluorescent secondary antibodies at room temperature for 2 hours, followed by DAPI counterstaining and mounting. Fluorescence imaging was performed using a fluorescence microscope (NCF950, Ningbo Yongxin Optics Co., Ltd.).

SCG Culture and Coculture

SCGs were dissected from postnatal day 0–3 mice under a stereomicroscope, cleaned in L-15 medium, cut into halves, and plated onto 24-well plates pre-coated with rat tail collagen (Yeasen, Cat# 40109ES76). Plates were incubated at room temperature for 1 h, air-dried, washed twice with PBS, and stored at 4 °C. Each well was seeded with 150 μL SCG culture medium containing high-glucose DMEM, 10% FBS, 1% penicillin/streptomycin, and 2.5 S mouse NGF (Thermo Fisher, Cat# 13257-019), then incubated at 37 °C with 5% CO_2. Medium was changed every 2 days. On day 4, mesenteric arteries from postnatal day 8 mice were isolated and added to the SCG culture. NP137 was supplemented into the medium at this stage to assess its effect during co-culture. After 3 days, samples were fixed in 4% PFA at 4 °C for immunofluorescence staining[4].

Enzyme-linked Immunosorbent Assay (ELISA)

Blood samples were collected from mice by the eyeball method and placed in a procoagulant tube. After resting at room temperature for 30 min, the samples were centrifuged at 3,000 rpm for 15 min to obtain serum. Ovaries were dissected immediately after *in vivo* imaging and frozen in liquid nitrogen for hormone and neurotransmitter detection. Serum and tissue levels of estradiol (E2), testosterone (T), dihydrotestosterone (DHT), luteinizing hormone (LH), follicle-stimulating hormone (FSH), progesterone (PROG), sex hormone-binding globulin (SHBG), NE, and E were quantified using ELISA kits (Longton Biological Technology, China), strictly following the manufacturer’s instructions.

Image Reconstruction and Structural Quantification

Digital images were processed and reconstructed using Imaris software (v 9.0, Bitplane). NIR-II-L time-lapse images of dynamic ovarian vasculature were analyzed with the Surface module for 3D rendering. For dual-channel NIR-II-L images, follicles were identified using the Spot module, and vasculature was reconstructed using the Filament tool. In optically cleared ovaries, follicles were detected with the Spot function, while vascular structures were identified with the Filament module. High-resolution 3D reconstructions of neurovascular coupling were performed using the Surface module to segment sympathetic nerves and vessels; their physical interaction was quantified using.

In addition, to quantify vessel diameters over time in NIR-II-L dynamic imaging, the ImageJ-based macro VasoMetrics was used[5]. This tool semi-automatically tracks single vessel segments across frames and calculates the average diameter and temporal changes.

Statistical Analysis

For quantitative assessments, all experiments, including NIR-II-L imaging, immunofluorescence staining, tissue clearing, and Western blotting were independently repeated at least three times. Data are presented as mean ± standard error of the mean (SEM). Statistical comparisons between two groups were performed using an unpaired Student's t-test, while one-way or two-way ANOVA followed by Tukey's post hoc test was used for comparisons among multiple groups. A P-value <0.05 was considered statistically significant. GraphPad Prism 9 (GraphPad Software, USA) was used for statistical analysis and graph preparation. Specific statistical tests, biological replicate numbers, and effect sizes are detailed in each figure legend.

Reference:

[1] Chen ZH, *et al*. NIR-II Anti-Stokes Luminescence Nanocrystals with 1710 nm Excitation for in vivo Bioimaging. *Angew Chem Int Ed Engl* 64, e202416893 (2025). https://doi.org/10.1002/anie.202416893

[2] Ramírez Hernández DA, *et al*. Role of the superior ovarian nerve in the regulation of follicular development and steroidogenesis in the morning of diestrus 1. *J Assist Reprod Genet* 37, 1477-1488 (2020). https://doi.org/10.1007/s10815-020-01787-6

[3] Matsumoto K, *et al*. Advanced CUBIC tissue clearing for whole-organ cell profiling. *Nat Protoc* 14, 3506-3537 (2019). https://doi.org/10.1038/s41596-019-0240-9

[4] Brunet I, *et al*. Netrin-1 controls sympathetic arterial innervation. *J Clin Invest* 124, 3230-3240 (2014). https://doi.org/10.1172/jci75181

[5] McDowell KP, Berthiaume AA, Tieu T, Hartmann DA, Shih AY. VasoMetrics: unbiased spatiotemporal analysis of microvascular diameter in multi-photon imaging applications. *Quant Imaging Med Surg* 11, 969-982 (2021). https://doi.org/10.21037/qims-20-920

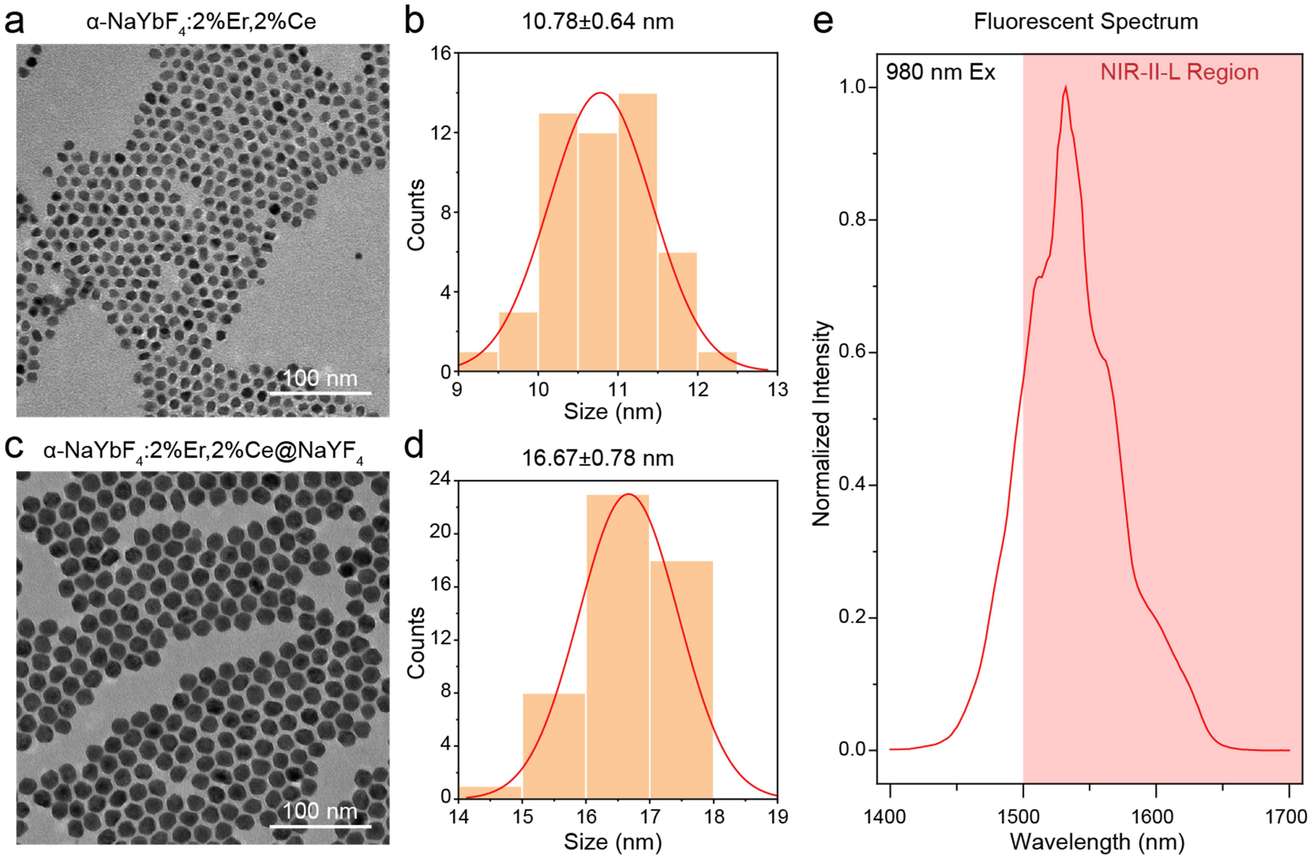

Fig. S1 Characterization of nanostructure and fluorescent properties of α-$NaYbF_4$:2%Er,2%Ce@$NaYF_4$ lanthanide downconversion nanocrystals.

a-b, TEM images and size distribution of α-$NaYbF_4$:2%Er,2%Ce nanocrystals. c-d, TEM images and size distribution of α-$NaYbF_4$:2%Er,2%Ce@$NaYF_4$ nanocrystals. e, Fluorescent spectrum of α-$NaYbF_4$:2%Er,2%Ce@$NaYF_4$ nanocrystals under 980 nm excitation.

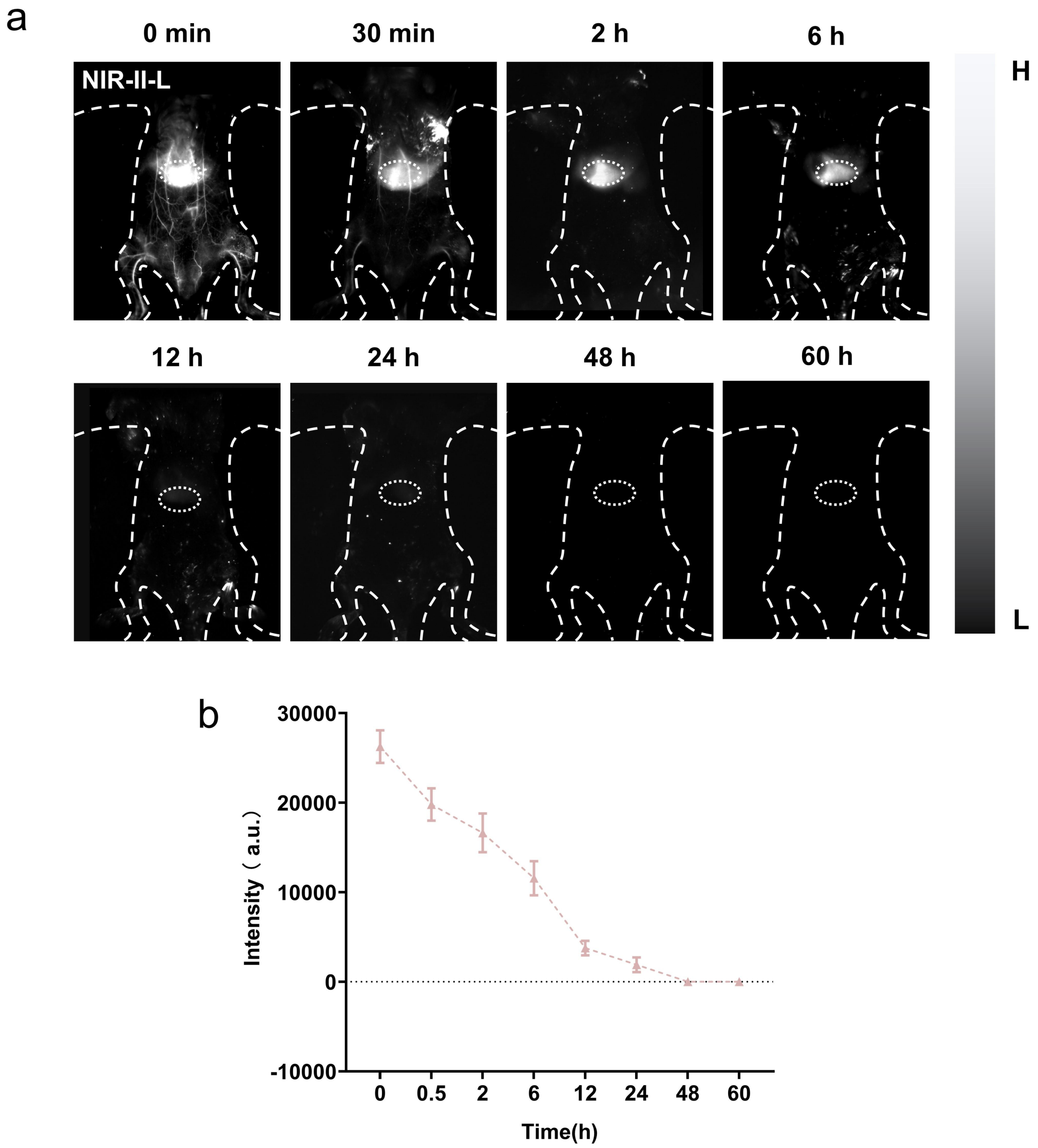

Fig. S2 *In vivo* circulation behavior of the nanoprobe characterized by time-series imaging. a, Representative whole-body NIR-II-L images acquired at different time points after intravenous injection of the nanoprobes (0 min to 60 h). b, Quantitative analysis of fluorescence intensity in the liver region over time.

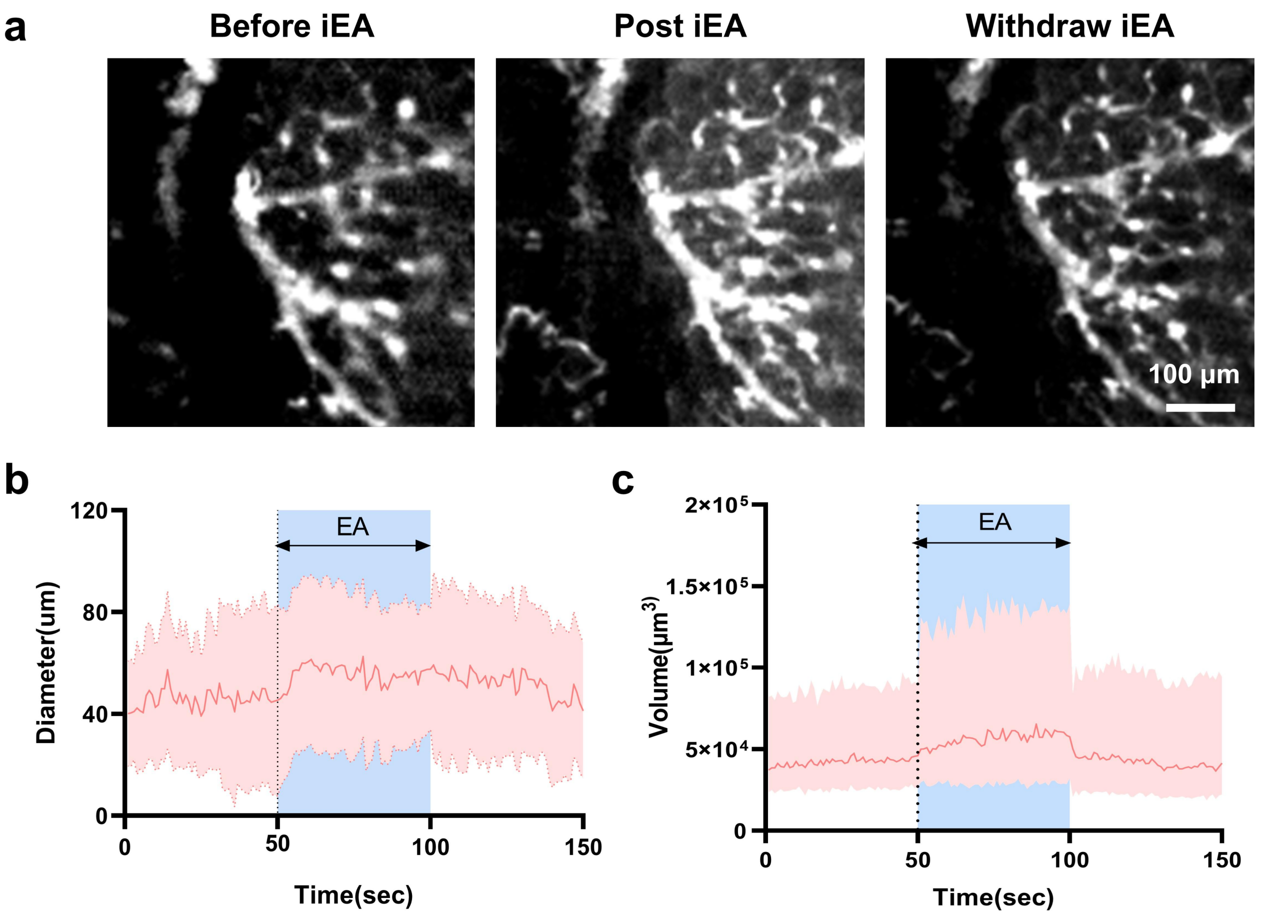

Fig. S3 Vascular dynamics during and after iEA. a,Representative NIR-II-L images of ovarian vasculature acquired Before iEA, Post iEA, and withdraw iEA are shown. b, Vascular diameter was quantified using full width at half maximum (FWHM) measurements, and (c) perfusion-related vascular volume was calculated over time.

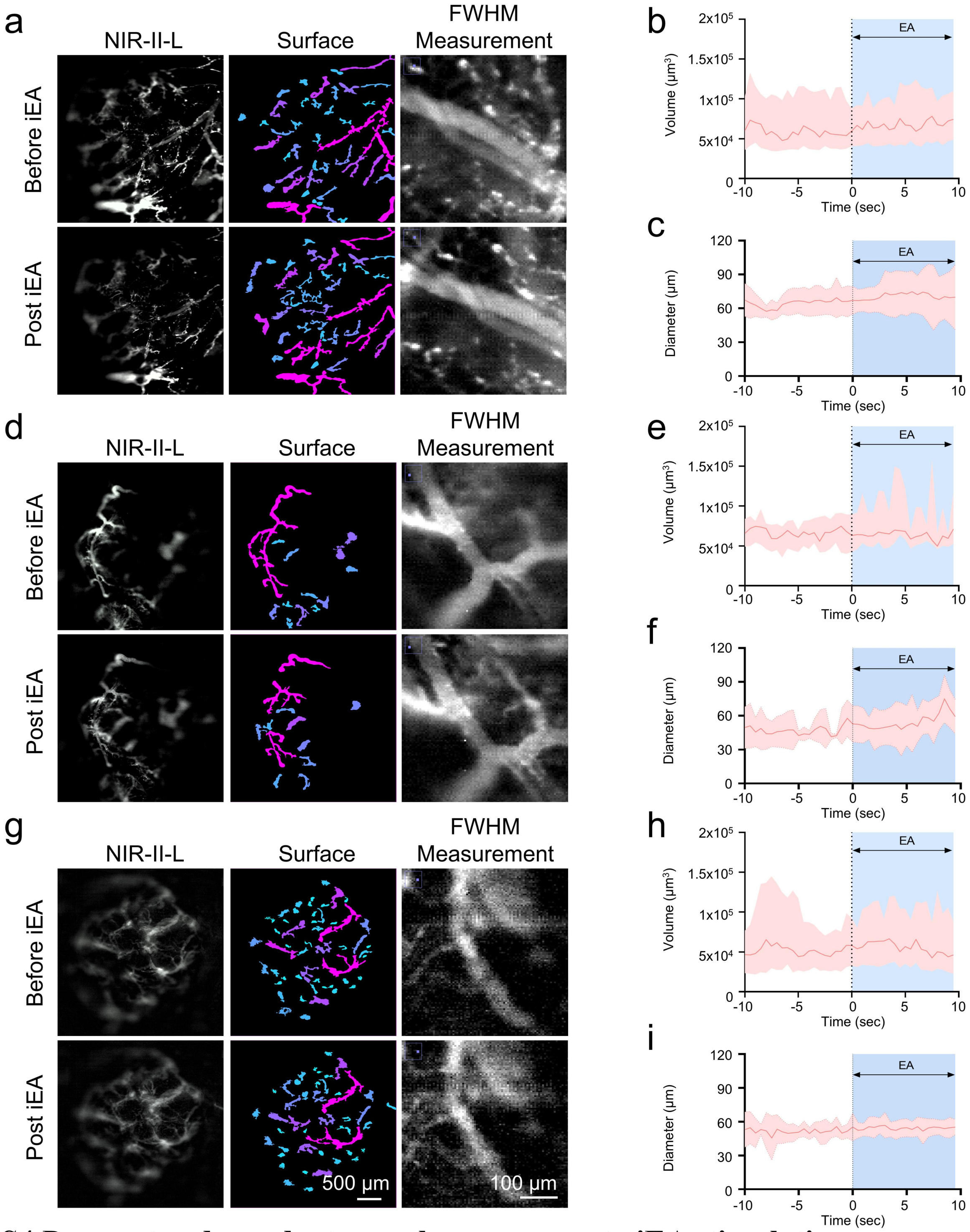

Fig. S4 Parameter-dependent vascular responses to iEA stimulation.
a, d, g, Representative NIR-II-L images, 3D surface reconstructions, and FWHM-based measurement of ovarian surface artery before and after iEA under different stimulation parameters (2 Hz 1 mA, 100 Hz 1 mA, 100 Hz 3 mA). b, e, h, Time course of total perfusion volume changes in ovarian region (n=1 per group), showing varying levels of increase upon stimulation onset (t=0). c, f, i, Time course of vessel diameter changes of the tracked vessel, revealing rapid and significant dilation after iEA under different stimulation parameters. Shaded areas represent mean±95% confidence interval.

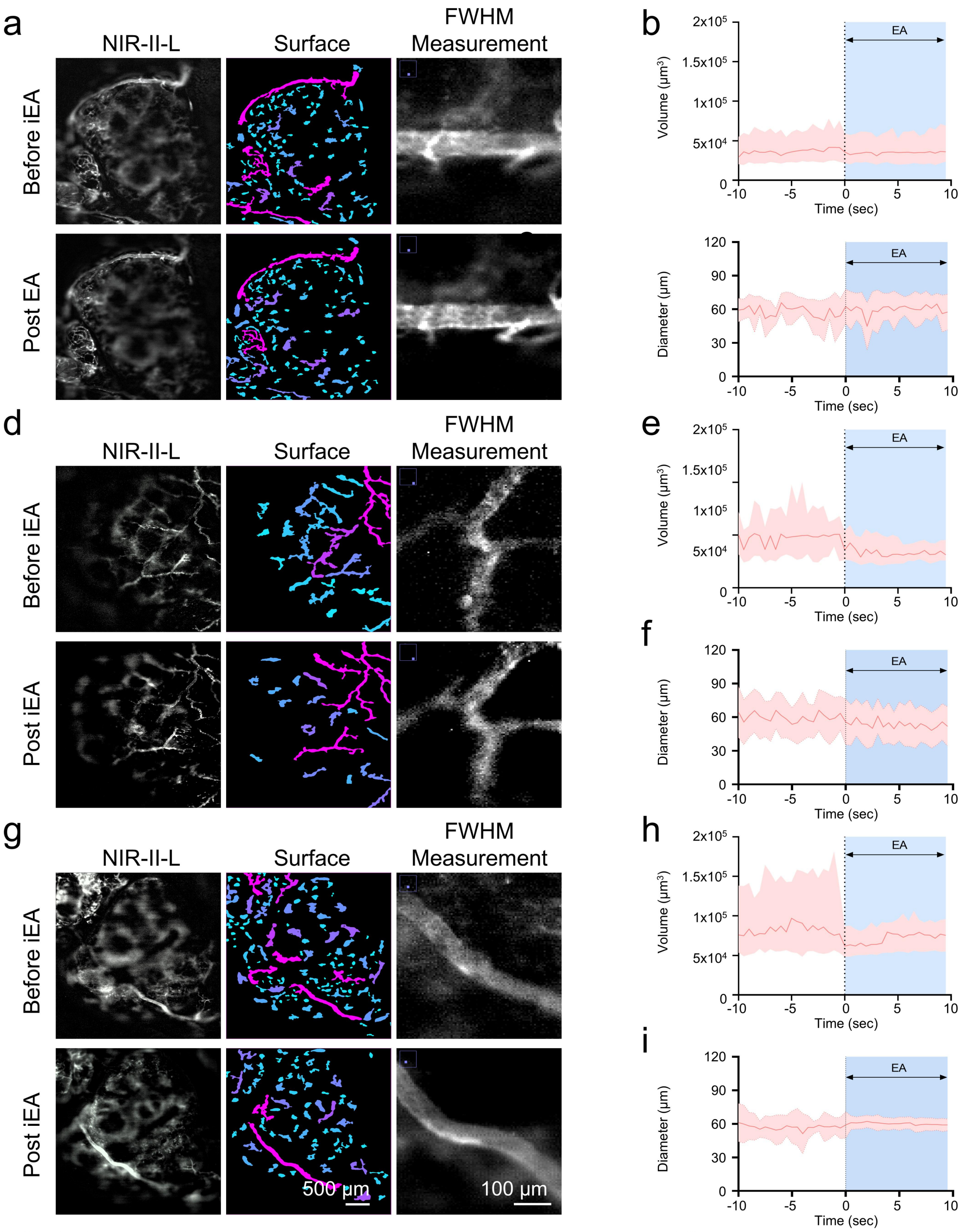

Fig. S5 iEA-induced vasodilation is both parameter-specific and acupoint-specific. a, d, Representative NIR-II-L images, surface reconstructions, and FWHM-based vessel measurements before and immediately after EA (2/100 Hz) at 1 mA (a) and 3 mA (d). b, e, Time course of perfusion volume following stimulation onset (t=0). c, f, Corresponding vessel diameter remained stable throughout the stimulation period. g, Changes in ovarian vessels before and after iEA stimulation of control acupoints. h, i, Perfusion and diameter measurements in the control group confirmed the acupoint-dependence of the iEA effect. Shaded areas represent mean±95% confidence interval.

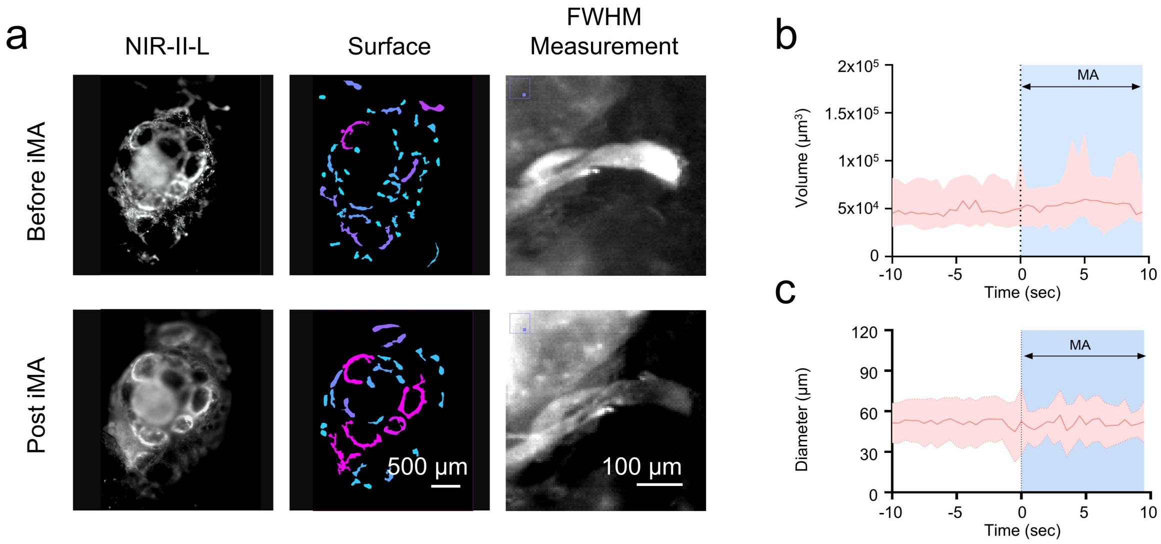

Fig. S6 Effect of iMA on ovarian perfusion and vascular diameter.
a, Representative NIR-II-L fluorescence images, vessel reconstructions, and FWHM-based diameter measurements of an ovarian surface artery before and after iMA at specific acupoints (SP6 and ST29). b, c, Time course of perfusion volume (b) and vessel diameter (c) following the onset of iMA (t=0 s). Shaded areas represent mean±95% confidence interval.

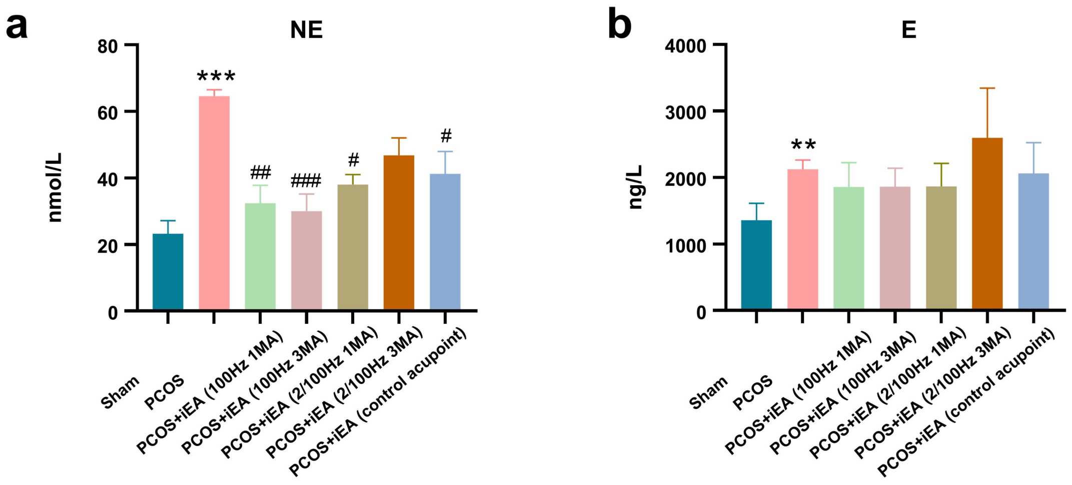

Fig. S7 Quantification of ovarian NE and E levels following iEA with different stimulation parameters.

a, NE concentrations in ovarian tissue across experimental groups, measured by ELISA. b, E concentrations in ovarian tissue under the same conditions. Data are shown as mean ± s.e.m., with individual values overlaid. Statistical analysis was performed using one-way ANOVA with Tukey's post hoc test. *P < 0.01, *** P < 0.001 vs. Sham; #P < 0.05, ##P < 0.01, ### P < 0.001 vs. PCOS.

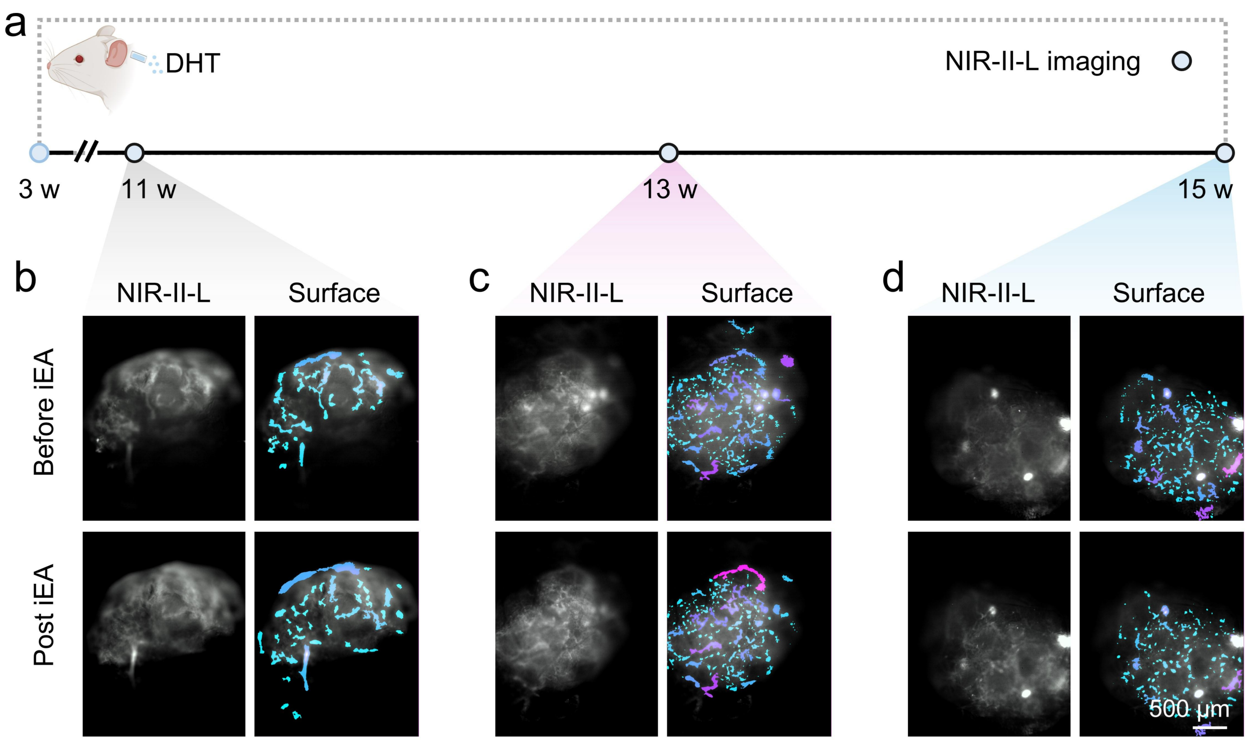

Fig. S8 NIR-II-L imaging of ovarian vasculature in PCOS mice without cEA intervention.

a, Experimental timeline of DHT-induced PCOS model and imaging schedule. NIR-II-L live imaging was performed at weeks 11, 13, and 15. b–d, Representative NIR-II-L fluorescence and surface vessel reconstructions of ovarian tissue from the same PCOS mouse at indicated time points. Each panel shows images before and immediately after imaging. Scale bars, 100 μm.

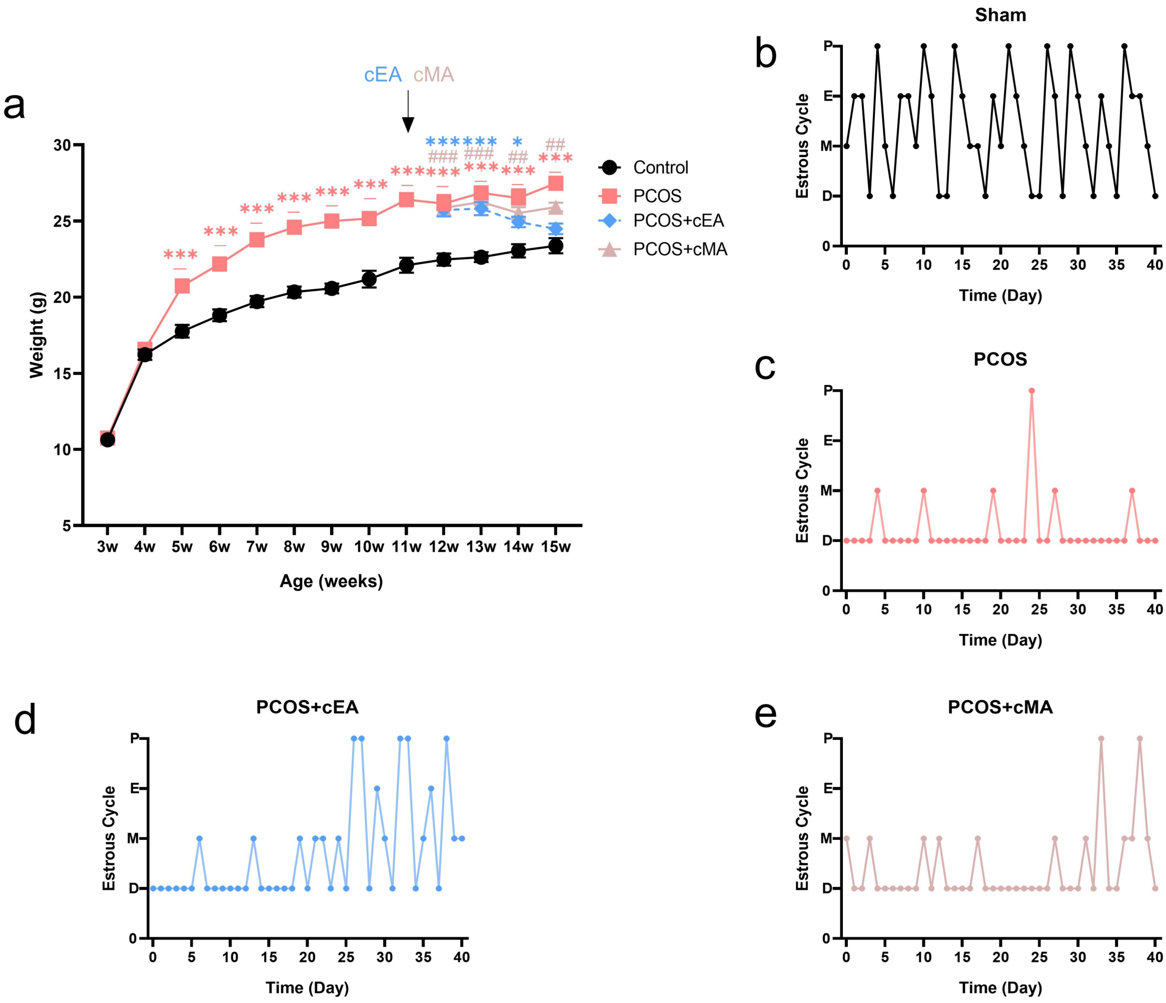

Fig. S9 Body weight and estrous cycle tracking in control and PCOS mice with or without cEA/cMA.

a, Body weight curves of mice from 3 to 15 weeks of age in control, PCOS, PCOS+cEA, and PCOS+cMA groups. cEA and cMA interventions were initiated at week 11.b–e, Representative estrous cycle patterns recorded over 40 consecutive days in Sham (b), PCOS (c), PCOS+cEA (d), and PCOS+cMA (e) groups. Vaginal smears were collected daily and classified into proestrus (P), estrus (E), metestrus (M), or diestrus (D). Data are presented as mean ± s.e.m. in panel a. Statistical analysis was performed using two-way ANOVA with Tukey's post hoc test.*P < 0.05, **P < 0.01, **P < 0.001 vs. Sham;#P < 0.05, ##P < 0.01, ###P < 0.001 vs. PCOS.

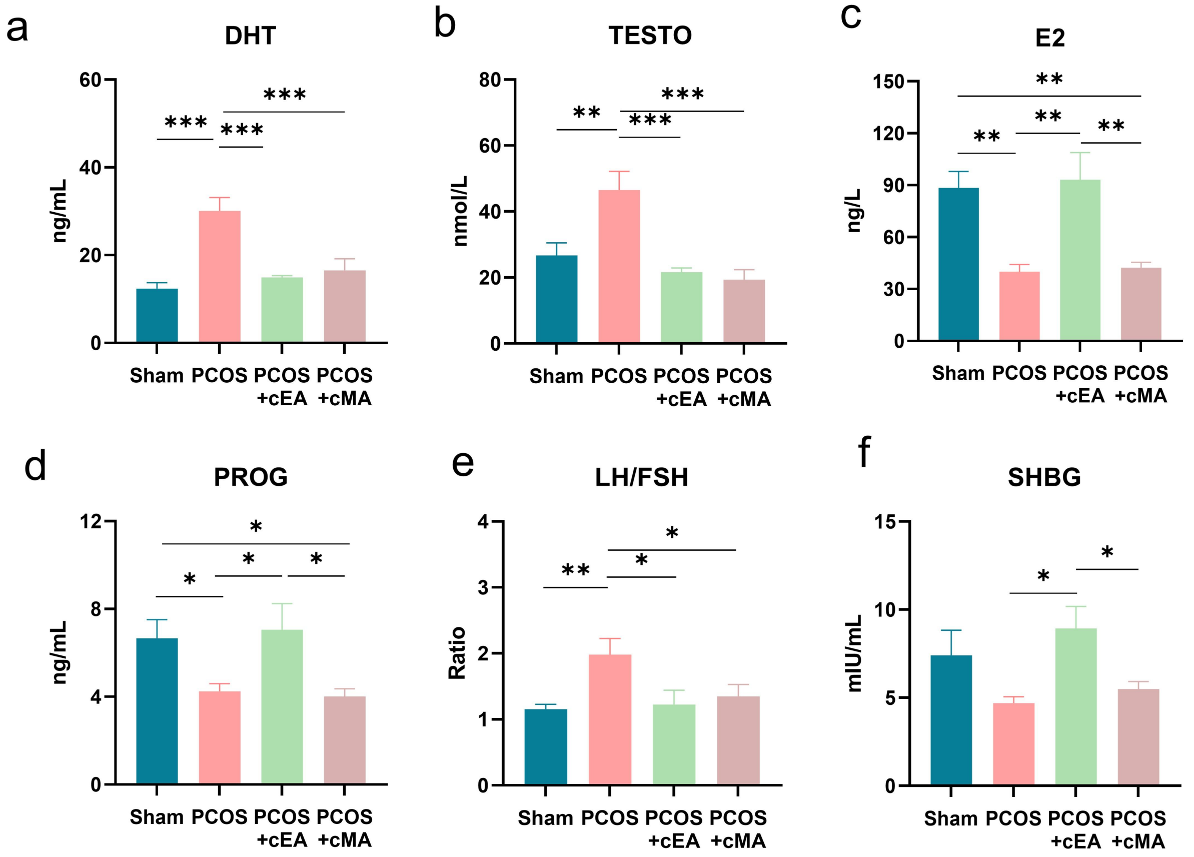

Fig. S10 Serum hormone levels in control and PCOS mice with or without cEA/cMA intervention.

a–f, Quantification of circulating hormone levels including: a, DHT, b, TESTO, c, E2, d, PROG, e, LH/FSH ratio, f, SHBG. Hormone levels were measured in serum collected from Sham, PCOS, PCOS+cEA, and PCOS+cMA groups. Data are presented as mean ± s.e.m. Statistical analysis was performed using one-way ANOVA with Tukey's post hoc test.*P < 0.05, **P < 0.01, **P < 0.001.

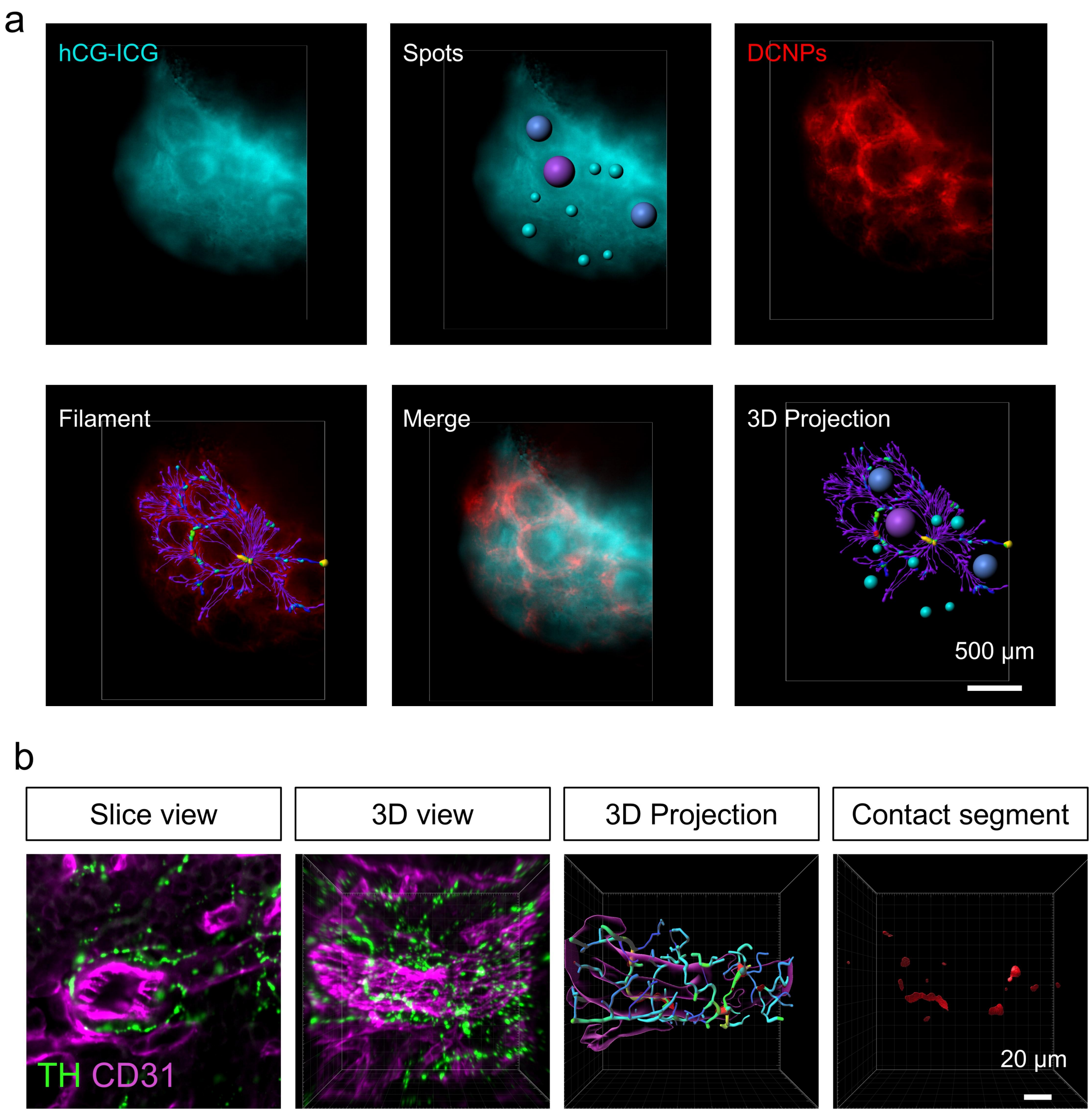

Fig. S11 3D visualization of the interaction of follicle-vascular-neural structures in PCOS ovaries after cMA intervention.

a, NIR-II fluorescence imaging of a whole ovary showing follicles (hCG–ICG, cyan), vasculature (DCNPs, red), and sympathetic nerves (filament tracing). Spots represent segmented follicles. Merge and 3D projection views illustrate the spatial configuration of these components. b, 3D immunofluorescence reconstruction of tyrosine hydroxylase (TH^+, green) and $CD31^+$ vasculature (magenta) from ovarian tissue. Slice, 3D, and projection views show the structural relationship between sympathetic terminals and vessels. Contact segment map indicates spatial proximity of TH^+ fibers to $CD31^+$ structures. Scale bars: 500 μm (a), 20 μm (b).

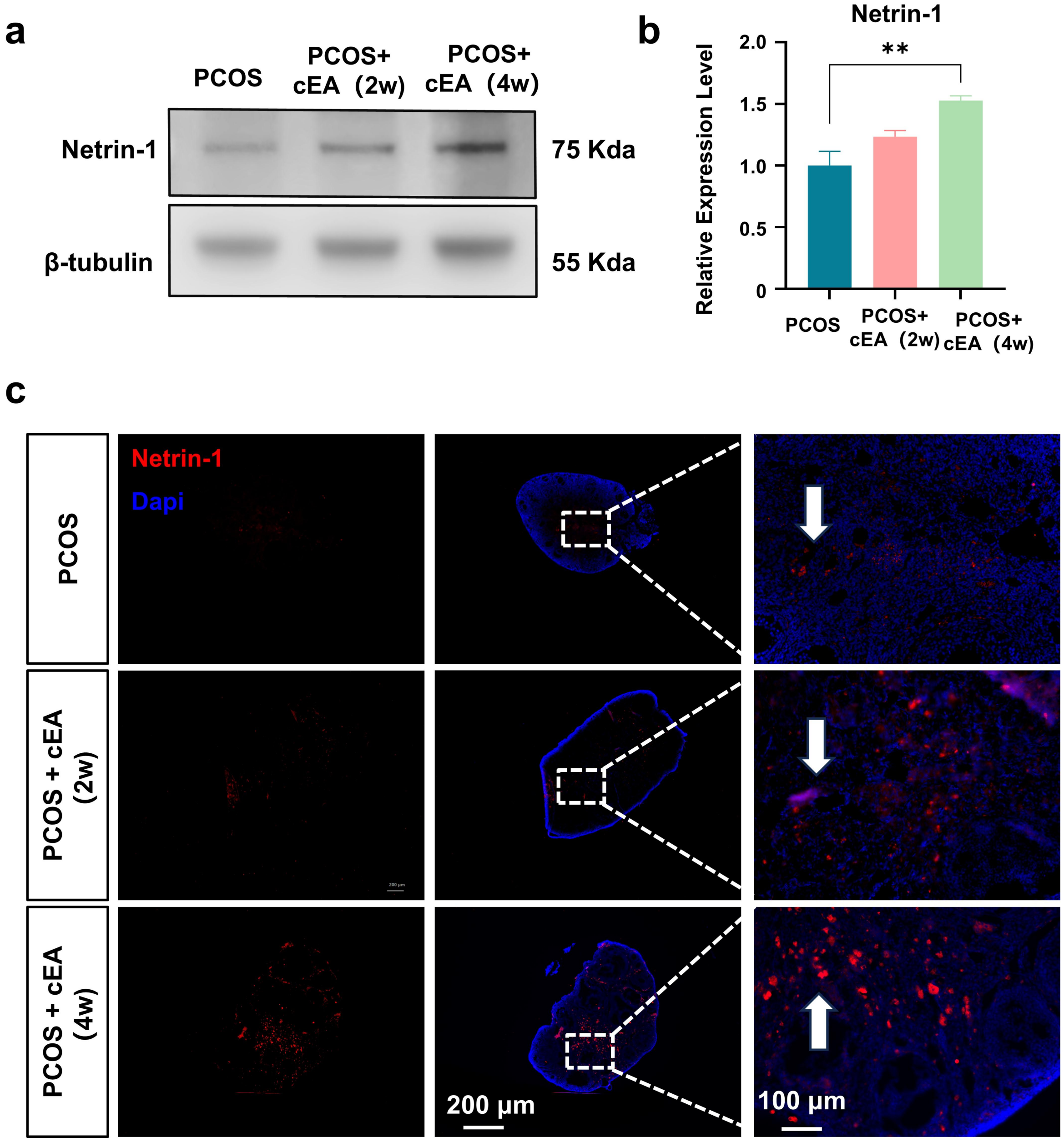

Figure S12. Time-dependent induction of Netrin-1 during cEA. a, Western blot analysis of ovarian Netrin-1 expression in PCOS mice and PCOS mice treated with cEA for 2 weeks and 4 weeks. b, Quantification of Netrin-1 protein levels (n=3 mice per group). Data are presented as mean ± SEM. One-way ANOVA with Tukey's post hoc test. $^{**}P < 0.01$.c, Representative whole-ovary immunofluorescence images showing Netrin-1 expression (red) in PCOS ovaries and ovaries treated with cEA for 2 and 4 weeks. Dashed boxes indicate regions shown at higher magnification. Arrows highlight increased Netrin-1 signal following prolonged cEA.

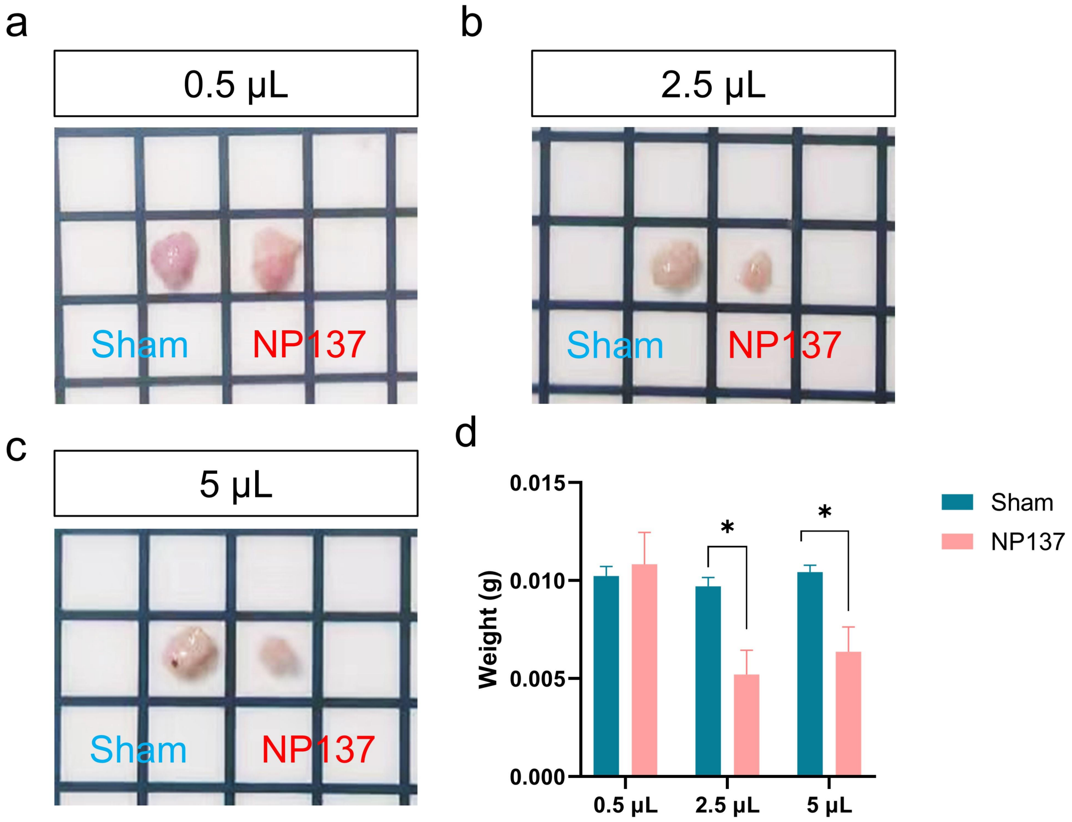

Fig. S13 Dose selection for NP137 injection in ovarian tissue.

a-c, Representative photographs of ovaries from Sham and Treated sides after local injection of NP137 at 0.5 μL (a), 2.5 μL (b), and 5 μL (c). d, Quantification of ovary weight following NP137 injection at different volumes (n=3 per group). Data are shown as mean±s.e.m. Statistical analysis was performed using paired t-test. $P < 0.05$.

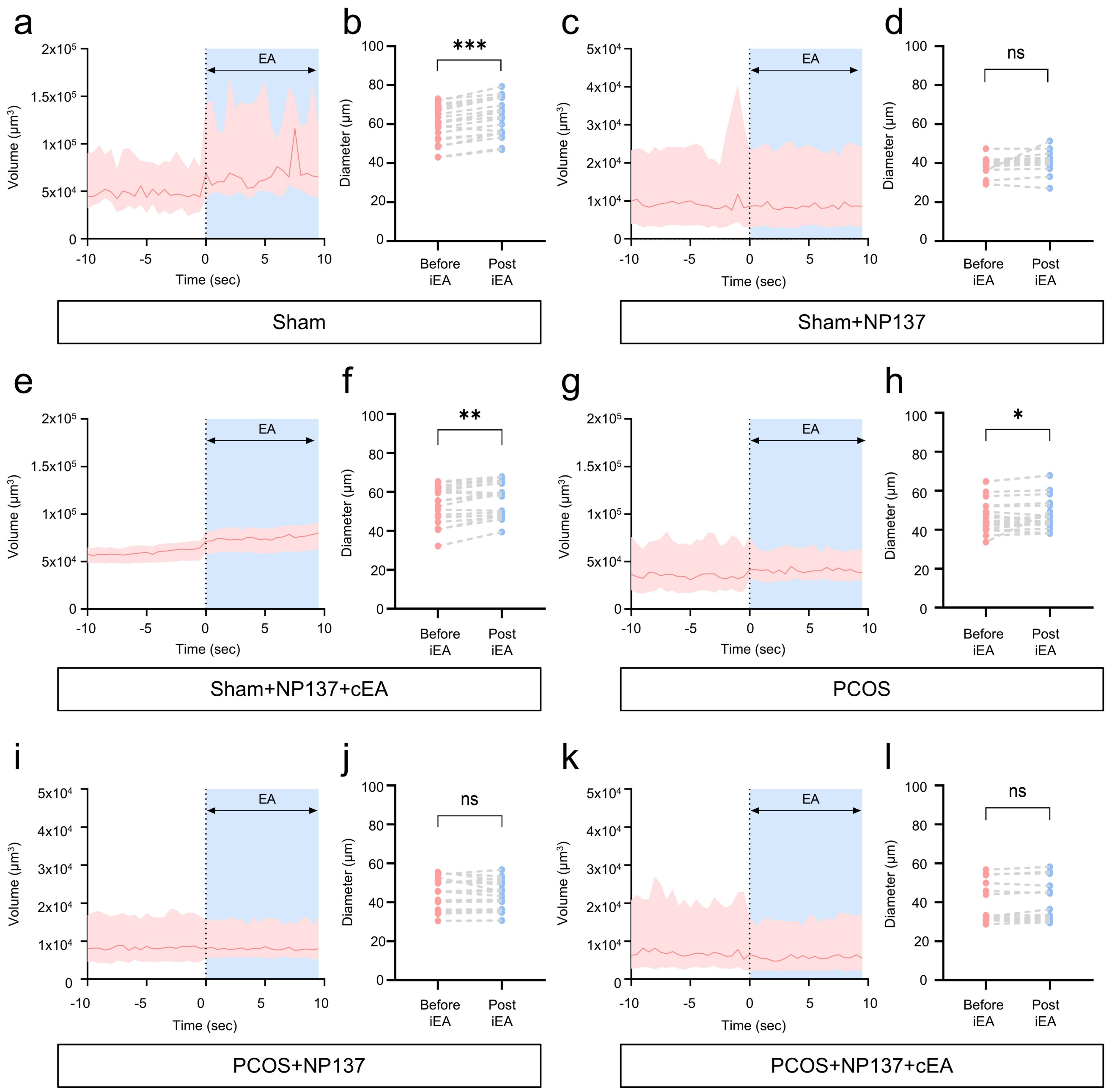

Fig. S14 Perfusion volume and vessel diameter changes before and after iEA across different experimental groups.

a-l, Time course of perfusion volume (a, c, e, g, i, k) and corresponding vessel diameter (b, d, f, h, j, l) in response to iEA in each groups. Perfusion was measured by NIR-II fluorescence signal intensity in the ovarian region. Vessel diameter was estimated by FWHM of a defined surface artery. Line plots (a, c, etc.) show group mean ± 95% confidence interval over time. Before–after plots (b, d, etc.) represent paired diameter values from 20 arterioles (3 mice per group) before and after iEA stimulation. Statistical comparisons were performed using the Wilcoxon matched-pairs signed rank test.*P < 0.05, **P < 0.01, **P < 0.001; ns, not significant.

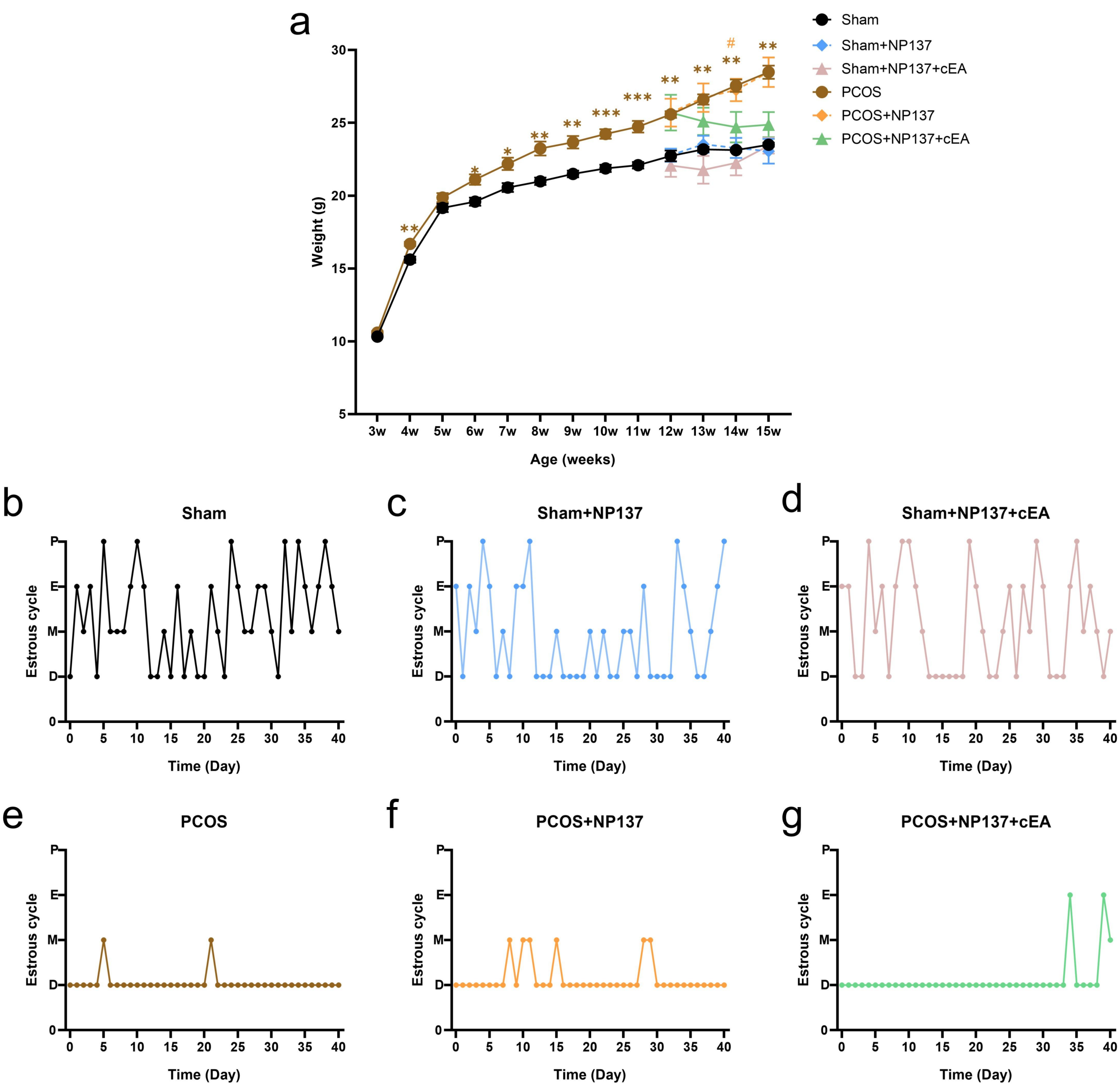

Fig. S15 Effects of NP137 and cEA on body weight and estrous cycles in Sham and PCOS mice.

a, Body weight curves from 3 to 15 weeks of age in Sham, Sham+NP137, Sham+NP137+cEA, PCOS, PCOS+NP137, and PCOS+NP137+cEA groups. b–g, Representative estrous cycle tracking over 40 consecutive days in each group. Vaginal smears were performed daily and stages classified as proestrus (P), estrus (E), metestrus (M), and diestrus (D). Data in a are shown as mean ± s.e.m. Statistical analysis used two-way ANOVA with Tukey's post hoc test.*$P < 0.05$, *$P < 0.01$ vs. Sham; #$P < 0.05$, ##$P < 0.01$ vs. PCOS.

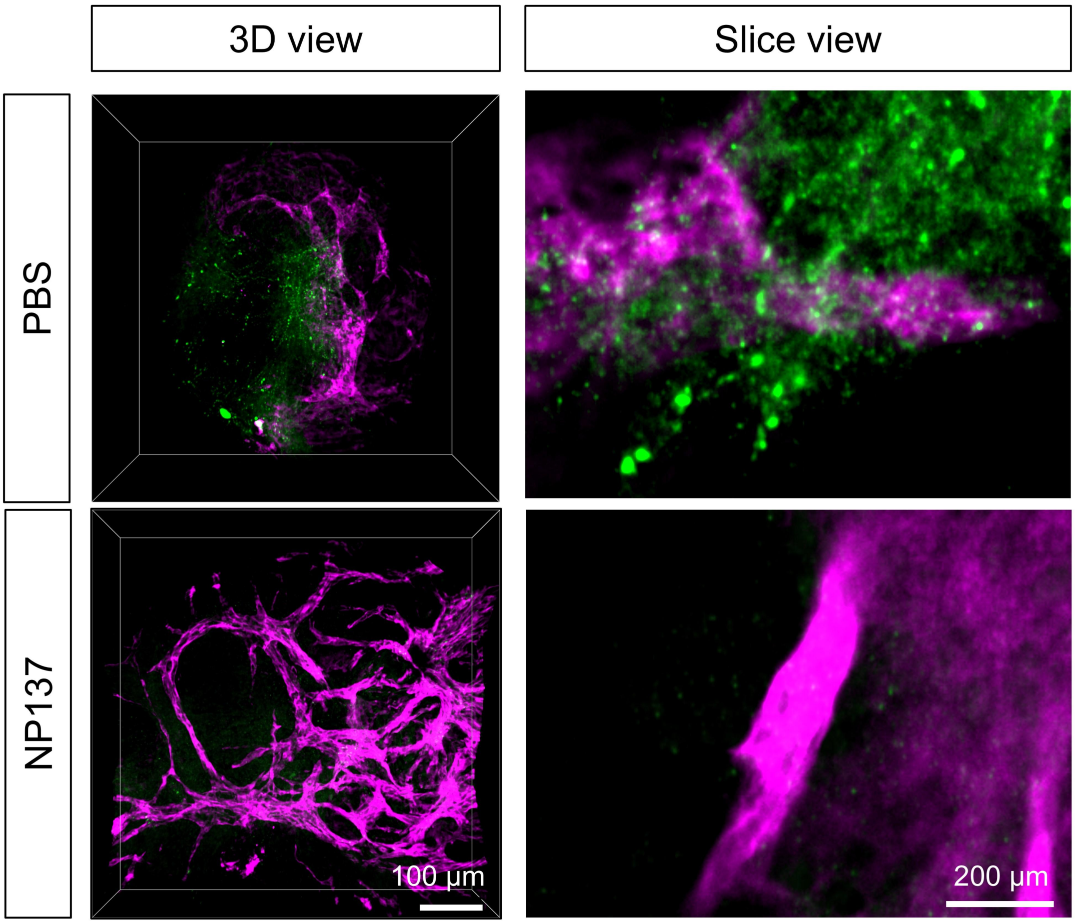

Fig. S16 Confocal imaging of TH[+] terminals and CD31[+] vessels in an ex vivo SCG–aorta co-culture model with or without NP137 treatment.

3D reconstructions (left) and optical slice views (right) of co-cultured sympathetic ganglia (TH[+], green) and aortic segments (CD31[+], magenta) under PBS (top) or NP137 (bottom) conditions. NP137 was added during the co-culture period. Scale bars: 100 μm (3D), 200 μm (slice).
